# Systematic Search for Recipes to Generate Induced Pluripotent Stem Cells

**DOI:** 10.1371/journal.pcbi.1002300

**Published:** 2011-12-22

**Authors:** Rui Chang, Robert Shoemaker, Wei Wang

**Affiliations:** Department of Chemistry and Biochemistry, University of California, San Diego, La Jolla, California, United States of America; Ecole Normale Supérieure, France

## Abstract

Generation of induced pluripotent stem cells (iPSCs) opens a new avenue in regenerative medicine. One of the major hurdles for therapeutic applications is to improve the efficiency of generating iPSCs and also to avoid the tumorigenicity, which requires searching for new reprogramming recipes. We present a systems biology approach to efficiently evaluate a large number of possible recipes and find those that are most effective at generating iPSCs. We not only recovered several experimentally confirmed recipes but we also suggested new ones that may improve reprogramming efficiency and quality. In addition, our approach allows one to estimate the cell-state landscape, monitor the progress of reprogramming, identify important regulatory transition states, and ultimately understand the mechanisms of iPSC generation.

## Author Summary

Converting somatic cells back to the stem cell state (called induced pluripotent stem cells or iPSCs) exemplifies the recent advancement of cellular reprogramming that holds great promise for developing regenerative medicine. Generation of iPSCs is often achieved by overexpressing three to four genes in somatic cells that are critical for regulating pluripotency. Developing optimal reprogramming recipe is a non-trivial task that requires significant effort. We present here a computational method that can facilitate discovery of effective recipes to generate iPSCs with high efficiency and better quality. In addition, our approach provides a new way to estimate the landscape in the cell-state space and monitor the trajectory of cellular reprogramming from a differentiated cell to an iPS cell. This work provides not only practical recipes for iPSC generation but also theoretical understanding of the reprogramming process.

## Introduction

Recent studies have shown that cellular reprogramming can be achieved by manipulating a small number of genes [1], [2]. This includes the generation of induced pluripotent stem cells (iPSCs) from somatic cells [3]–[6] and the conversion of one differentiated cell type directly to another (transdifferentiation) [7]–[10]. These findings hold enormous promise for disease modeling and regenerative medicine. However, the reprogramming efficiency is often low and the mechanistic process of reprogramming remains largely unknown. In addition, recent studies have shown that iPSCs generated by the present recipes are different from embryonic stem cells (ESCs) on such as exonic mutation [11], copy number variation [12], chromosome aberration [13], epigenetic [14] and immunogenicity [15] deviation from ESCs. Resolving these problems is essential to realize the full potential of therapeutics based on cellular reprogramming.

One of the possible reasons for the above problems of the current iPSCs is that reprogramming recipes utilize suboptimal combinations of reprogramming factors. Most studies to date first identify a pool of candidate reprogramming factors (around 20) that are differentially expressed in two cell types [3], [4], [8]–[10]. If overexpression of all these factors can convert one cell type to another, sequential removal or adding of these factors one at a time is then conducted to find whether a factor is crucial for reprogramming, through which a minimal set of reprogramming factors is found. Such a procedure requires a significant amount of effort and the greedy search for combinations of the preselected factors does not necessarily find the optimal reprogramming recipe.

Efficiently finding optimal reprogramming recipe requires a systematic search for any perturbation (not necessarily limited to a preselected set of factors) to the cell that can achieve the most effective reprogramming. Achieving this goal requires *de novo* prediction of phenotypes, i.e. predicting the consequences (reprogramming) of perturbations. Recent studies showed that gene expression can be predicted based on TF binding information [16]–[18] and prediction of phenotypes based on the topology of genetic networks is feasible [19]–[27]. These studies illustrated the great potential of systems biology approach in understanding fundamental principles of biology and developing therapeutic treatments. However, none of these existing methods was designed for or applied to searching for optimal reprogramming recipe. Therefore, new systems biology methods are still needed for such purpose.

Having a mechanistic picture of cellular reprogramming requires a comprehensive understanding of the biological system of interest. There are numerous theoretical studies on cell fate decision based on differential equations, but they often focus on simplified circuits that do not embody the molecular details required to understand the mechanisms of how cellular reprogramming is achieved [28], [29]. Epigenetic landscape [30] has been used to explain cell differentiation during development and cell fate reprogramming [31]–[33]. The landscape concept has been widely appreciated in protein folding/binding [34], [35] and more recently in genetic network analysis [36]–[41]. Particularly, recent studies have provided quantitative models for theoretical understanding of development from a landscape viewpoint [40], [41]. However, the current methods of calculating network landscapes are time consuming and thus limited to small networks (often <20 genes) [38], which cannot illustrate the mechanistic procedure of iPS reprogramming or transdifferentiation.

In this study, we present a new approach to systematically search for optimal reprogramming recipes and to provide mechanistic insights in reprogramming human cells from the perspective of the cell-state potential landscape, as encouraged by our previous work on the model organism budding yeast [42], [43]. Based on a network curated from literature that includes the major regulatory interactions known in human embryonic stem cells (hESCs), we developed a method to make *de novo* predictions of gene expression changes upon perturbations such as overexpression or knockdown of genes. Our predictions correlated well with knockdown experiments in hESCs. In addition, our model allows efficient calculation of the probability of any cell state for a large network. These features made it possible to systematically search for optimal reprogramming recipes and to establish the cell-state landscape. Without knowledge of any successful reprogramming recipes, the recipes we identified included several experimentally confirmed ones that were only recently published [44]. Our study fills the gap between theoretical and experimental studies on iPSCs, and illustrates a framework to facilitate experimental design and mechanistic interpretation of the experimental observations.

## Results

### Genetic network regulating hESC

**Figure 1:**
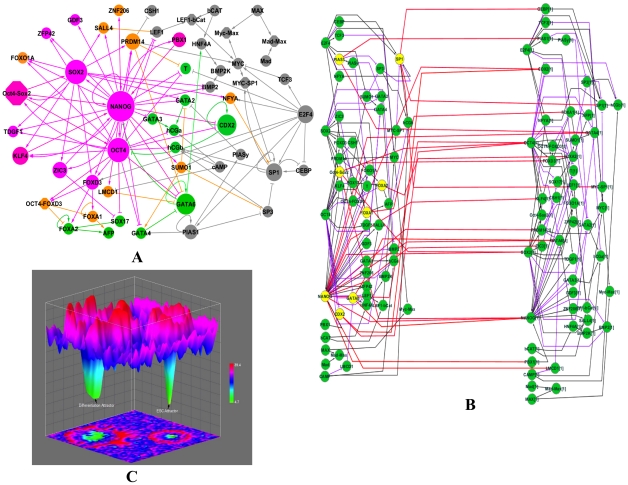
Epigenetic landscape. (**A**)**. Genetic network regulating self-renewal and differentiation of hESC.** Active and repressive regulations are represented by arrow and bar links, respectively. Node size is proportional to a node's total degree (sum of incoming- and outgoing-degrees). The hESC and differentiation markers are colored in pink and green, respectively. The genes positively regulated by hESC markers are colored in orange. (**B**)**. 2-time slice Bayesian Networks (2TBN) model of genetic network in hESC.** The 2TBN consists of 2 slices of Bayesian networks and each slice contains a complete set of 52 nodes in the original network. All the loops are deconvolved into inter-slice edges (in red), which represent the regulations between the regulators in the first time slice (interface proteins, colored in yellow) and the regulated genes in the second time second time slice. The outgoing interface consists of NANOG, SP1, Oct4-Sox2, CDX2, PIAS1, GATA6, FOXA2, and FOXA1. The purple and black edges represent intra-time slice down regulating and up regulating effects, respectively. (**C**)**. Illustration of the cell-state potential landscape.** The color represents the potential of the cell state. The higher the potential, as represented on the z-axis, indicates a smaller probability of that particular cell state. X and Y coordinates specify unique cell states.

We first collected evidence from literature and manually constructed a genetic network involved in regulating pluripotency and hESC differentiation (Figure 1A, Table S1 and S2 in Text S1). We did not employ any data mining or bioinformatics methods in constructing the network to avoid false regulatory interactions. We started with a set of marker genes of pluripotency and differentiation lineages (Table S3 in Text S1) and extensively searched the literature for regulatory paths between any pair of genes. This constructed network is composed of direct regulatory interactions between 52 nodes, including the three key regulators of ESC (Oct4, NANOG and Sox2), six protein complexes (Oct4-Sox2, Oct4-Foxd3, LEF1-bCat, Mad-Max, Myc-Max, Myc-Sp1) as well as marker genes for the differentiation lineages (Table S3 in Text S1). Considering the difference between human and mouse ESCs, we focused on regulatory interactions that have direct evidence in the hESC. The completeness and correctness of this network were partially confirmed by its capability to correctly predict the gene expression changes upon Oct4 knockdown (see below).

### Estimating the network landscape

As shown in previous studies, estimating the landscape of a network requires calculating the probability of each cell state. To accomplish this task, we considered each protein in the network as either active or inactive, i.e. each node is a binary variable. We then used a dynamic Bayesian network (DBN) [45] to model the feedback loops in the network. The probability of each node represents how likely the protein is active.

DBN simulates the evolving stochastic characteristics of the network via temporal organization of a 2-time slice Bayesian network (2TBN). In order to transform the cyclic hESC network to a DBN, we need to break all cyclic regulations and unroll the network into a series of acyclic graphs (2TBN), in which interface proteins either emit or receive feedbacks in the original network. We employed a searching procedure to identify interface proteins such that the unrolled 2TBN not only reduced the computational complexity but also preserved biological meaningful links (see Methods and Text S1 for details) (Figure 1B). As the DBN evolved to its steady state, information was updated and propagated through these interface proteins from the current time slice to the next using the interface algorithm [45].

To parameterize the DBN of the genetic network, ideally one should learn the parameters from a large set of temporal functional data that reflects the regulations between proteins in the network. Due to the lack of such data in hESCs, we designed a knowledge-based model that converted functional links in the curated genetic network to mathematically meaningful parameter constraints (see Text S2 for details) [46], [47]. Next, we exploited the Monte Carlo Markov Chain (MCMC) method to sample DBN parameter values based on these constraints. Each set of parameter samples formed an instance of the DBN model. All the model instances were averaged to conduct DBN inference, which allowed calculation of the joint probability of all the proteins in the network given specific evidence. Compared with the previous approaches of calculating network landscapes using either Boolean network or differential equations, our model is much more efficient. Compared with the conventional DBN model, our method avoids determination of the large number of parameters by reverse-engineering. Our study showed that this model was sufficient for reprogramming recipe discovery (see below).

To compute the network landscape, we need to consider all possible extracellular conditions. Because the exact transduction of extracellular signals to TFs is largely unknown in hESCs, we chose an alternative approach by manipulating the expression levels of the three key hESC regulators, which are Oct4, Sox2 and NANOG. This mimics the effects of extracellular conditions for maintaining pluripotency or inducing differentiation. We calculated the joint probabilities of all the nodes in the network when setting Oct4/Sox2/NANOG to various activity levels and then summed these probabilities to estimate the network landscape (see Text S2). The obtained landscape (Figure 1C) represents the steady state probability of the system. The landscape of the system at a certain time during differentiation can be obtained by specifying the activity of the three key hESC regulators.

We found two states with significantly higher probabilities than the rest of the states and they respectively correspond to the hESC and differentiated states (Figure 1C), as defined by the activity of the 22 marker proteins (Table S3 in Text S1). When all the 11 ES markers are active (1) and when all the 11 differentiation markers are inactive (0), the network represents a hESC state; the differentiated state is defined as the opposite activity composition of these 22 markers. These two states are separated by barrier states with smaller probabilities. These barriers prevent transformation between cell types by noise. This result is similar to the epigenetic landscape proposed to describe the differentiation process of ESCs [30]–[33]. To our knowledge, this is the first landscape generated for a genetic network of a reasonably large size (52 nodes) that can reflect the molecular details of regulation on self-renewal and differentiation of hESC.

### Predicting phenotypes (gene expression) change upon perturbations

In order to search for reprogramming recipes, we first need to show that our model could predict the consequences of cellular perturbations. Knockdown of master regulators Oct4 or NANOG was recently performed in hESCs and gene expression changes were measured by either microarray (Won et al., submitted and [48]) or PCR [49]. These datasets were used to test our model. In the framework of the DBN, we modeled the knockdown of a gene by clamping its activity value to a specific level and then conducting inference. After the DBN converged, the ratio between the probability of each node in the perturbed and the undisturbed hESC state was calculated. This ratio was compared with the experimental gene expression change. The undisturbed hESC state in this study was hypothesized to be the joint probability of all nodes when the hESC master regulators Oct4, NANOG and Sox2 were all clamped to an active state (see Methods and Text S2).

**Figure 2:**
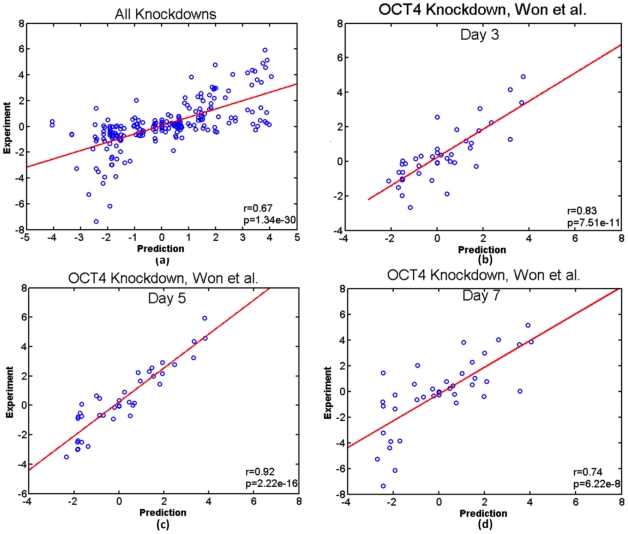
Pearson correlation coefficient (r) and p-value (p) between predicted and experimental gene expression changes (in log2). (A) A scatter plot of predicted and experimental gene expression changes of all OCT4(Won et al., submitted and [54]) and NANOG knockdown [55] experiments; (B), (C) and (D) Scatter plots of day 3, 5 and 7 after knocking down OCT4 using an episomal vector(Won et al., submitted).

When we pooled six OCT4 and NANOG knockdown experiments together in hESC, the DBN predicted values correlated well with the experimental measurements (Pearson correlation coefficient = 0.6731 and p-value = 1.34*10^−30^) (Figure 2). This correlation varied per individual experiment and fell in a range between 0.55 and 0.92 (Figure 2 and S1 in Text S1). For example, a comparison between predicted and experimental gene expression changes after day 3, 5 and 7 of OCT4 knockdown yielded Pearson correlation coefficients of 0.83, 0.92 and 0.74, respectively. Note that our predictions were made solely based on the genetic network topology, without any other functional data. The accuracy of *de novo* phenotypic predictions based on cellular perturbations demonstrated that our DBN model can reliably search for iPS reprogramming recipes.

### Reprogramming recipes and mechanisms

We used two types of criteria to judge the success of a predicted recipe: (1) Gene expression similarity to the ESC and (2) Reprogramming efficiency. The predicted state of reprogrammed cells should achieve an expression signature similar to hESCs (see Text S1 and S2). This similarity was measured by the root mean square deviation (RMSD) and the Pearson and Spearman rank correlation coefficients between the reprogrammed and the hESC expression levels (joint probabilities). The reprogramming efficiency was defined as the percentage of cells predicted to be in differentiated states (11 hESC markers off and 11 differentiation markers on) that could be reprogrammed to any attractor representing the ES state (11 hESC markers on and 11 differentiation markers off). To simulate the heterogeneity of the differentiated cell states, we started from 100,000 randomly initialized states of the 30 non-markers in the network, then appropriately clamped the proteins involved in the specific reprogramming recipe, and finally evolved all proteins' states until convergence by following their maximum a posterior (MAP) pathways in DBN evolution (see Text S2).

We calculated the expression similarity and reprogramming efficiency for all 163,185 possible combinations of overexpressing 4 out of the 46 individual genes in the network. We found that most of the overexpression combinations did not achieve reprogramming as indicated by a reprogramming efficiency of zero. Indeed, only 962 recipes had an efficiency greater than 0. We found that efficiency was not necessarily correlated with expression similarity (Figure S2 in Text S1). The expression similarity measurement reflected how similar the final state was to the hESC state by comparing expression levels between all the 52 nodes. Reprogramming efficiency only checked 22 nodes in the network (11 hESC markers and 11 differentiation markers). A high reprogramming efficiency did not thusly guarantee a final cell state that was similar to a hESC state. Therefore, an optimal recipe should have both high efficiency and high expression similarity to the hESC state (low RMSD and high Pearson and Spearman correlation coefficients).

Among the 962 recipes with an efficiency larger than 0, we found three experimentally validated recipes using OCT4 (O), SOX2 (S), KLF4 (K), c-MYC (M) or PRDM14 (P) Encouraged by this observation, we further confirmed the success of the 3-factor (OSK) and 5-factor (OSKMP) experimental recipes (Table 1). The predicted reprogramming efficiencies were consistent with the experimental observations: OSKMP is more efficient than OSKM, OSKP is more efficient than OSK [44]. When either OCT4 or SOX2 was removed from a recipe, the reprogramming efficiency became zero, which was also observed in the experiments that leaving out either OCT4 or SOX2 could not generate iPSC [44]. In addition, both reprogramming efficiency and gene expression similarity measurements clearly distinguished those successful recipes from the experimentally unsuccessful ones (Table 1). It is worth noting that our predicted recipes solely relied on the network topology and did not use any information from Chia et al. [44] and our method [46], [47], [50] was developed before the publication of [44].

**Table 1:** Experimentally validated reprogramming recipes.

Reprogramming Recipes	Experimental iPSC generation [44]	Predicted iPSC Reprogramming Efficiency^a^	Expression similarity to the ESC state^b^		
			RMSD	Pearson (r)	Spearman (r)
OCT4_SOX2_MYC_PRDM14	Yes	0.357106	0.089769	0.955399	0.966317
OCT4_SOX2_KLF4_PRDM14	Yes (7 folds of OSK efficiency) [44]	0.356406	0.053386	0.988467	0.989982
OCT4_SOX2_KLF4_MYC_PRDM14	Yes (3.5 fold of OSKM efficiency) [44]	0.339366	0.086431	0.958194	0.967652
OCT4_SOX2_KLF4_MYC	Yes	0.231418	0.086810	0.959018	0.967399
OCT4_SOX2_KLF4	Yes	0.210998	0.055700	0.989700	0.988800
OCT4_MYC_KLF4	No	0	0.147900	0.888600	0.917300
SOX2_MYC_KLF4	No	0	0.162500	0.871400	0.909100
OCT4_MYC_PRDM14	No	0	0.374400	−0.006200	−0.103700
SOX2_MYC_PRDM14	No	0	0.427400	−0.338900	−0.386800

a Reprogramming efficiency reflects whether the recipe can convert a differentiated cell to an iPSC.

b Expression similarity to the hESC state reflects how similar the reprogrammed state is to the hESC state measured by root-mean-square-deviation (RMSD) and Pearson and Spearman correlation coefficients between gene expression of the reprogrammed state. The hESC state is simulated by clamping OCT4, Sox2 and NANOG to 1.

The consistency between our predictions and the experimental observations is encouraging. Our observation of many possible successful reprogramming recipes is consistent with the epigenetic landscape concept [30]–[33], which also shows that a large number of transition routes exist between two cell types. To confidently select for new reprogramming recipes, we first ranked the 962 4-factor recipes with efficiency>0 plus the experimental 3-factor (OSK) and 5-factor (OSKMP) recipes using each of the four criteria (reprogramming efficiency, RMSD, Pearson and Spearman). An average rank score was then computed to rank these recipes. Next, based on the individual distributions of RMSD, Pearson and Spearman of these recipes (Figure S3(a)–(c) in Text S1), we set a cutoff of 3-standard deviations for each criterion. 113 4-factor recipes (including OSKP) plus the 3-factor (OSK) recipe passed the cutoff (Dataset S1). Based on the averaged rank score, the top 10 recipes under each composition of master regulators are listed in Table 2.

**Table 2:** Top 10 candidate reprogramming recipes in each composition of master regulators.

OCT4_SOX2	OCT4_NANOG	OCT4_SOX2_NANOG
OCT4_SOX2_LMCD1_PRDM14	OCT4_NANOG_GDF3_ZFP42	OCT4_SOX2_NANOG_KLF4
OCT4_SOX2_PBX1_ZIC3	OCT4_NANOG_PBX1_ZFP42	OCT4_SOX2_NANOG_PBX1
OCT4_SOX2_PBX1_PRDM14	OCT4_NANOG_FOXO1A_PBX1	OCT4_SOX2_NANOG_GDF3
OCT4_SOX2_KLF4_PBX1	OCT4_NANOG_FOXO1A_GDF3	OCT4_SOX2_NANOG_TDGF1
OCT4_SOX2_FOXO1A_PBX1	OCT4_NANOG_GDF3_PBX1	OCT4_SOX2_NANOG_ZFP42
OCT4_SOX2_GDF3_PBX1	OCT4_NANOG_FOXO1A_TDGF1	OCT4_SOX2_NANOG_FOXO1A
OCT4_SOX2_PBX1_TDGF1	OCT4_NANOG_FOXO1A_ZFP42	OCT4_SOX2_NANOG_ZNF206
OCT4_SOX2_PBX1_ZFP42	OCT4_NANOG_KLF4_ZFP42	OCT4_SOX2_NANOG_PRDM14
OCT4_SOX2_LMCD1_PBX1	OCT4_NANOG_PRDM14_ZFP42	OCT4_SOX2_NANOG_ZIC3
OCT4_SOX2_PBX1_ZNF206	OCT4_NANOG_FOXO1A_KLF4	OCT4_SOX2_NANOG_FOXD3

**Figure 3:**
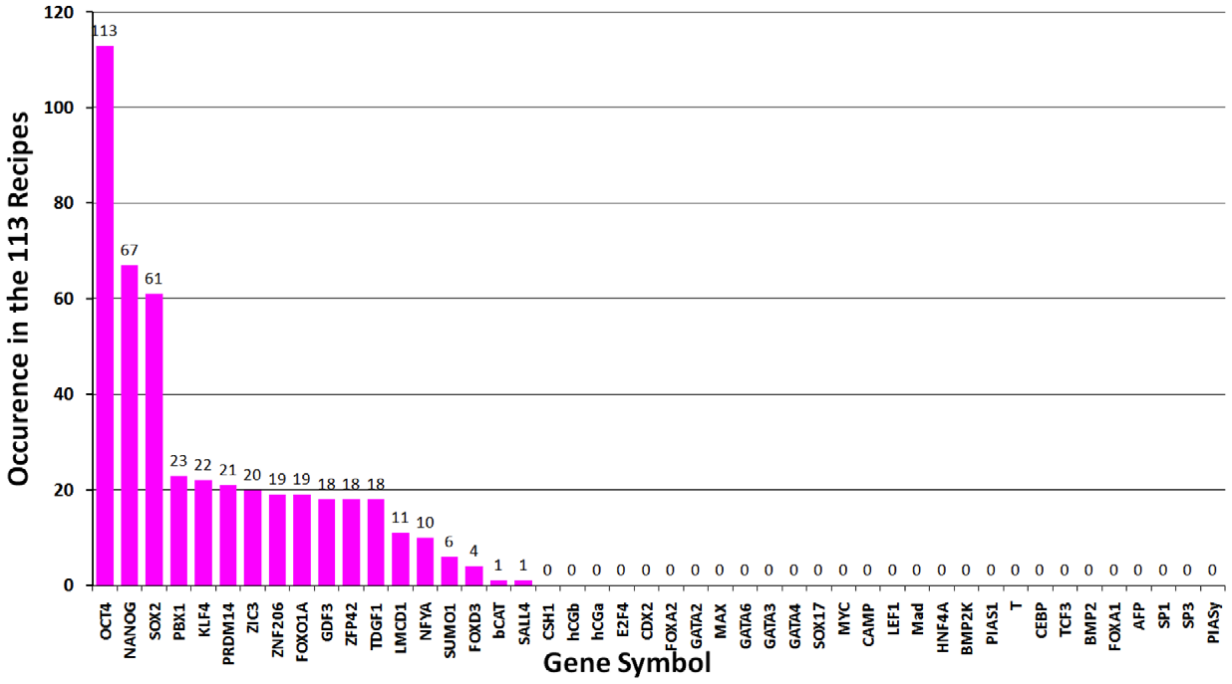
Occurrence of proteins in the 113 4-factor candidate reprogramming recipes.

All candidate reprogramming recipes (Dataset S1 and Table 2) contained at least two of the three master regulator genes (OCT4, SOX2, NANOG). In fact, all recipes without at least two master regulators had a reprogramming efficiency of zero. OCT4 was indispensible in generating iPSCs while SOX2 and NANOG were mutually replaceable (Figure 3). KLF4 and PRDM14 could substantially increase reprogramming efficiency. In addition, KLF4, c-MYC or PRDM14 could be substituted by other factors such as ZIC3, PBX1 or LMCD1. Although the mechanisms of how these additional factors function in iPSC generation are unclear, we speculate that their importance is due to either their positive feedbacks to the three master regulators such as PBX1 and ZIC3's activation on NANOG, or repression of the differentiation genes such as LMCD1's repression of GATA6, which is a repressor of NANOG (Figure 1). Other than the OSN, we found KLF4, PBX1, ZIC3 and PRDM14 occurred more than 20 times in the 113 recipes (Figure 3). All 16 combinations of these 4 genes with OSN were included in the candidate list (Dataset S1 and Table 3).

**Table 3:** Representative candidate reprogramming recipes.

OCT4_SOX2	OCT4_NANOG	OCT4_SOX2_NANOG
OCT4_SOX2_PBX1_PRDM14	OCT4_NANOG_PRDM14_ZIC3	OCT4_SOX2_NANOG_PRDM14
OCT4_SOX2_PBX1_ZIC3	OCT4_NANOG_KLF4_ZIC3	OCT4_SOX2_NANOG_ZIC3
OCT4_SOX2_KLF4_PBX1	OCT4_NANOG_PBX1_ZIC3	OCT4_SOX2_NANOG_KLF4
**OCT4_SOX2_KLF4_PRDM14**	OCT4_NANOG_KLF4_PRDM14	OCT4_SOX2_NANOG_PBX1
OCT4_SOX2_PRDM14_ZIC3	OCT4_NANOG_KLF4_PBX1	
OCT4_SOX2_KLF4_ZIC3	OCT4_NANOG_PBX1_PRDM14	

The experimentally confirmed recipe (OSKP) is highlighted in bold.

We examined whether the knockdown of individual genes would further enhance efficiency of overexpression-only reprogramming recipes. We took the five experimentally confirmed overexpression recipes as the templates and added an additional single gene knockdown. Among the 230( = 5×46) recipes, only the knockdown of GATA6 could significantly increase reprogramming efficiency for every experimental recipe without deteriorating the gene expression similarity (Table 4). This may be due to GATA6's repression of NANOG, which when attenuated would improve NANOG expression in a reprogramming recipe. The knockdown of GATA2 also increased the reprogramming recipe efficiency but it did not always increase the expression similarity (Dataset S2). Over 60% of single gene knockdowns including differentiation markers such as SOX17, GATA3, T, CDX2, hCGa, hCGb, AFP, and FOXA2 had negligible effect on reprogramming efficiency of the original recipes (see Figure S4 in Text S1). Surprisingly, knockdown of GATA4, which is a differentiation marker, prevented reprogramming, which might be due to its downregulation of GATA6. On the other hand, knockdown of non-marker genes including PRDM14 and LMCD1 also deteriorated iPSC generation. Our analyses illustrated the importance of choosing the right combination of perturbations and the usefulness of our computational modeling to quickly screen a large number of recipes.

**Table 4:** Effect of GATA6 knockdown on reprogramming.

Experimental Reprogramming Recipes (Over- expression & GATA6 Knockdown)	Predicted iPSC ReprogrammingEfficiency	Expression similarity to the ESC state		
		RMSD	Pearson (r)	Spearman (r)
OCT4_SOX2_MYC_PRDM14&GATA6	0.613516	0.081913	0.961799	0.965876
OCT4_SOX2_KLF4_PRDM14&GATA6	0.616624	0.044800	0.989263	0.987023
OCT4_SOX2_KLF4_MYC_PRDM14&GATA6	0.586480	0.079915	0.964070	0.968269
OCT4_SOX2_KLF4_MYC&GATA6	0.627800	0.079739	0.964028	0.966130
OCT4_SOX2_KLF4&GATA6	0.570686	0.044417	0.990064	0.987042
OCT4_MYC_KLF4&GATA6	0	0.113600	0.895640	0.923445
SOX2_MYC_KLF4&GATA6	0	0.122500	0.881400	0.933425
OCT4_MYC_PRDM14&GATA6	0	0.314400	−0.003200	−0.100370
SOX2_MYC_PRDM14&GATA6	0	0.387400	−0.328900	−0.342112

**Figure 4:**
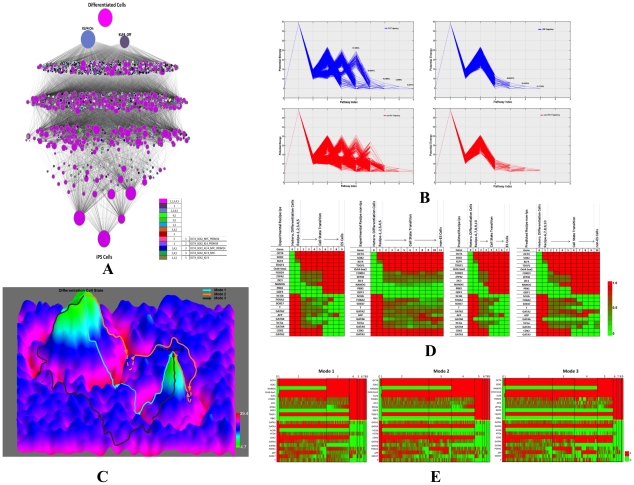
Reprogramming procedure. (**A**)**. State transitions during iPSC generation by the five experimentally confirmed recipes (overexpression of OCT4, SOX2, KLF4, PRDM14, or MYC).** Each recipe was sampled 100,000times. For illustrative purposes, each cell state was only defined by the 22 marker genes. The node size is proportional to the dynamical flux, which is defined as the number of paths going through the node. The color of nodes indicates the dynamical path of which experimentally validated recipes going through this node. Note that different reprogramming paths consist of a variable number of states, i.e. different paths pass through a variable number of nodes. (**B**)**. Potential changes along the reprogramming paths by the five experimental recipes (left) and experimental recipes plus knockdown of GATA6 (right).** Upper (blue lines) and lower (red lines) panels respectively show the paths that converged to the hESC and non-hESC attractors. The numbers indicate the percentages of initial states converged to the hESC attractors at the corresponding steps. X-axis values specify simulation step numbers. (**C**)**. Schematic illustration of the three modes of reprogramming by the 5 experimental recipes (** **Table 1** **) on the potential landscape (upside down of** **Figure 1** **).** The z-axis specifies the potential energy of a cell state and each cell state is specified by x- and y-coordinates. Mode 1 represents the most efficient (shortest) reprogramming paths spent least steps, mode 2 represents (moderate length) reprogramming pathways spent one extra steps in the valley, and mode 3 represents the least efficient (longest) reprogramming pathway with two extra steps in the valley. (**D**)**. Gene expression changes of marker genes during reprogramming.** As the simulation progresses, the hESC marker genes are down regulated while the differentiation marker genes are up regulated. The gene expression colors represent averaged values at each step. This analysis was based on experimentally validated reprogramming recipes. (**E**)**. Expression of marker genes in the three modes of experimentally validated reprogramming recipes.** Each column represents a unique state. The width of each simulation step (from initialization at step 0 to the converged joint probability at step 9) during reprogramming is proportional to the number of distinct cell states at that step: the larger the width, the more distinct cell states.

To better understand the mechanisms of iPSC generation, we monitored how reprogramming proceeded. We first calculated the potential of each cell state and found the most probable route (MAP path) starting from a differentiated state to its converged final state (see Methods and Text S2). The reprogramming progresses of the experimentally confirmed recipes are shown in Figure 4A as an illustration. Consistent with the landscape concept, there were many reprogramming paths and all of them went through two barriers, one surrounding the differentiation attractors and another near to the hESC attractors. Different paths had a variable number of steps between the two barriers that defined three modes of reprogramming. Interestingly, if GATA6 was knocked down, all the reprogramming paths had a decreased duration between the two barriers and quickly found the converged state (Figure 4B and C). We also monitored the gene expression changes of the 22 marker genes during reprogramming. Compared with the successful iPSC paths, those converged to non-ES states showed relatively higher expression of GATA6 and GATA2 (Figure 4D and E).

**Figure 5:**
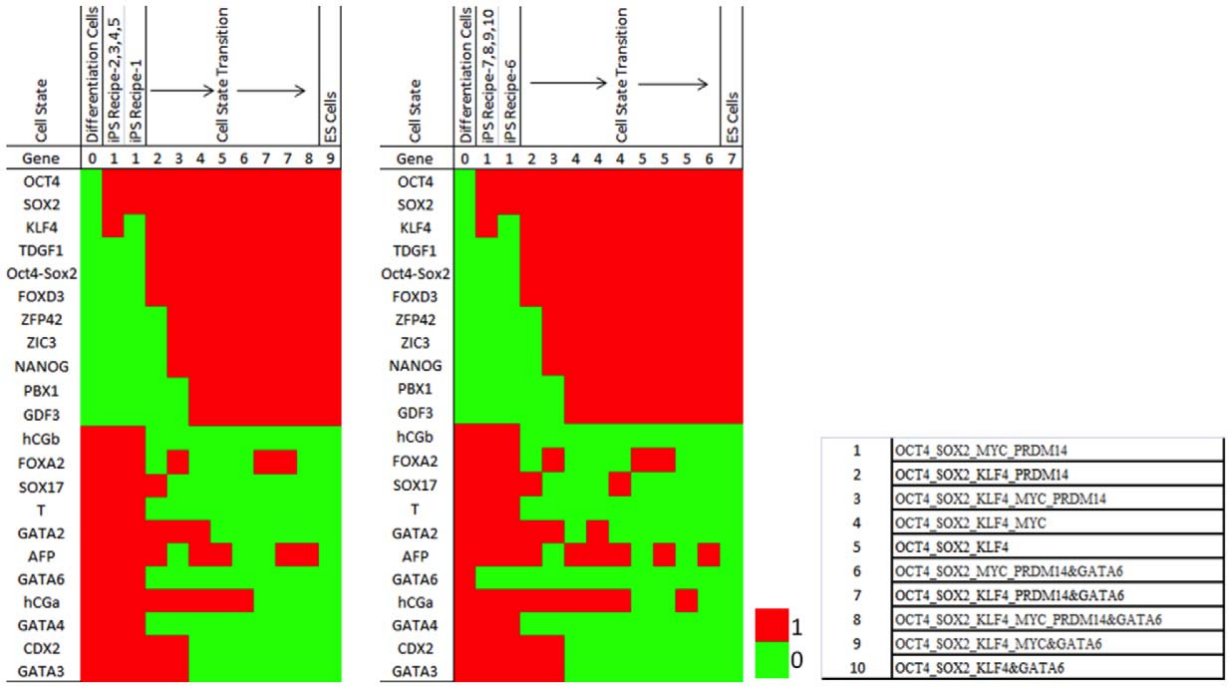
Transition states in the reprogramming. The cell states with larger than 1000 dynamical flux at each simulation step of reprogramming are shown for the 5 experimental recipes and the experimental recipes plus GATA6 knockdown.

Despite the difference of evolving steps, the three modes of reprogramming showed similar temporal gene expression patterns (Figure 4E, Table 5 and 6), which suggested there might be common transition states during reprogramming. We counted the number of initial states that passed a specific state during their evolution and this number was defined as the dynamical flux of the cell state. Using an arbitrary cutoff of 1000 for the flux, we found several states as highly probable transition states during reprogramming. Figure 5 shows the transition states for the 5 experimental recipes with and without GATA6 knockdown. A prominent pattern emerged from these states was that GATA6 was partially repressed by overexpressed OCT4 and this repression activated NANOG. NANOG's activation then led the consequent activation of pluripotent genes and repression of differentiation genes. Knockdown of GATA6 would enhance this regulation to facilitate reprogramming.

**Table 5:** Transition states (expression levels) during reprogramming using the five experimental recipes.

Gene	DifferentiationAttractor	DifferentiationBarrier	Valley at step(2,3,4)	hES Barrier Climb at 3,4,5	hES Valley at step 4,5,6,7,8	hES Attractor at step 4,5,6,7,8
OCT4	0	1	1	1	1	1
SOX2	0	1	1	1	1	1
NANOG	0	0	0	1	1	1
Oct4-Sox2	0	0	1	1	1	1
KLF4	0	0.5	1	1	1	1
FOXD3	0	0	0.6161	0.5172	1	1
ZIC3	0	0	0.5094	0.4721	1	1
ZFP42	0	0	0.5248	0.4893	1	1
GDF3	0	0	0	0	1	1
TDGF1	0	0	1	1	1	1
PBX1	0	0	0	0	1	1
GATA2	1	1	0.4883	0.4893	0.2075	0
GATA3	1	1	1	1	0	0
hCGb	1	1	0	0	0	0
hCGa	1	1	0.4415	0.368	0.3774	0
CDX2	1	1	1	1	0	0
GATA4	1	1	0.6475	0.5043	0	0
GATA6	1	1	0.3577	0	0	0
FOXA2	1	1	0.5927	0.6352	0.3774	0
AFP	1	1	0.5779	0.441	0.4528	0
SOX17	1	1	0.4119	0.3509	0.3774	0
T	1	1	0.4313	0.4506	0	0

**Table 6:** Transition states (expression levels) during reprogramming using the five experimental recipes plus GATA6 knockdown.

Gene	Differentiation Attractor	Differentiation Barrier	Valley at step 2	hES Barrier at step 3	hES Valley at step 4,5,6	hES Attractor at 7
OCT4	0	1	1	1	1	1
SOX2	0	1	1	1	1	1
NANOG	0	0	0	1	1	1
Oct4-Sox2	0	0	1	1	1	1
KLF4	0	0.5	1	1	1	1
FOXD3	0	0	0.547	0.51	1	1
ZIC3	0	0	0.5022	0.4843	1	1
ZFP42	0	0	0.5098	0.4943	1	1
GDF3	0	0	0	0	1	1
TDGF1	0	0	1	1	1	1
PBX1	0	0	0	0	1	1
GATA2	1	1	0.5186	0.4915	0.16	0
GATA3	1	1	1	1	0	0
hCGb	1	1	0	0	0	0
hCGa	1	1	0.5153	0.4843	0.4	0
CDX2	1	1	1	1	0	0
GATA4	1	1	0.546	0.4858	0	0
GATA6	1	0	0	0	0	0
FOXA2	1	1	0.5033	0.7137	0.4	0
AFP	1	1	0.5405	0.3604	0.6	0
SOX17	1	1	0.4978	0.463	0.32	0
T	1	1	0.5142	0.4843	0	0

## Discussion

In this study, we curated a genetic network composed of direct regulatory interactions that regulate self-renewal and differentiation of hESC. We developed a machine learning method to make *de novo* predictions of gene expression changes upon perturbations to the network. Our predictions were validated by a strong correlation between predicted and experimental values in OCT4 and NANOG knockdown experiments. We conducted a systematic search for new recipes that could achieve reprogramming. In addition to recovering several experimentally confirmed recipes, our study provided a wealth of new recipes that serve as a guide for improving experimental iPSC generation. Our theoretical analyses suggested knocking down additional genes, such as GATA6, would further enhance experimentally known reprogramming recipes. Since our framework is general, it is also applicable to other cellular reprogramming such as transdifferentiation.

We defined two criteria to assess whether a recipe could achieve reprogramming: reprogramming efficiency and gene expression similarity to hESCs. We noticed that the calculated reprogramming efficiencies were much higher than the experimentally observed ones of around 0.01% to 0.1%. There are several possible reasons. In our modeling, overexpression or knockdown of genes is 100% efficient but in reality the efficiency of such perturbations is imperfect. In addition, our modeling only considers whether the recipe can induce pluripotency. It did not consider the proliferation efficiency of the reprogrammed cell. Therefore, the calculated values should be taken as an upper bound of the reprogramming efficiency. When calculating gene expression similarity to hESCs, we used the gene expression profile of the state with active OCT4, SOX2 and NANOG genes to represent the normal hESC. Once the upstream signaling pathways of these master regulators are defined, a better modeling strategy would incorporate environmental signals into the network and let extracellular signals control the activities of the master pluripotency regulators.

Although the present results successfully recovered experimentally validated recipes and suggested new ones, many aspects of our approach can be improved. With the advancement of technologies to manipulate hESCs, new regulatory interactions will be quickly discovered, which will expand the genetic network used in this study. This is expected to improve the accuracy of gene expression prediction and recipe identification. With the availability of additional data such as temporal gene expression of self renewal or induced differentiation, our DBN model can be further trained by incorporating such data to improve/expand the network to better consider proliferation efficiency (for reprogramming efficiency) and the resemblance (for reprogramming quality) of the reprogrammed cells to the natural targeted cells [43].

In summary, this study and our previous work on yeast [43] suggest a new systematic strategy to find recipes for a desired reprogramming task. Namely, a genetic network regulating the original and target cell types is constructed from existing knowledge or learned by incorporating experimental data. A mathematical model such as DBN or probabilistic Boolean network can be used to conduct inference on gene expression or other phenotypes based on the network, which allows exhaustive or comprehensive search of perturbations (recipes) that can convert the phenotype representing the original cell type to that representing the target cell type. This work is a proof-of-concept study that forms the foundation of applying such strategy to find effective recipes to achieve any cellular reprogramming with satisfactory efficiency and quality.

## Methods

To search for reprogramming recipes, we need to make *de novo* predictions of the phenotypic consequences of perturbations to a genetic network. We use expression levels of all the genes in the network to represent phenotypes, i.e. ES or differentiation state. We chose dynamic Bayesian network (DBN) to model the curated hESC network (Figure 1) that contain many feedback loops. Prediction of consequences of a perturbation is an inference problem in DBN. Genomic data, either perturbation (gene knockdown or overexpression) or temporal gene expression data, that are conventionally used to train the parameters or learn the structure of the DBN, are very limited in hESC. Therefore, we chose a method based on the constraint imposed by the network structure on the parameter space to conduct inference. We showed that this method achieved a satisfactory performance on predicting gene expression changes upon perturbation in the hESC (Figure 2 and S1 in Text S1). Our model also allows efficient calculation of the potential landscape in the cell state space and monitoring the reprogramming progress in this landscape. We outline our approach below and the details of the model can be found in the Text S2.

### Constraint-based Qualitative Knowledge-DBN (QK-DBN) model

We constructed DBN model, referred as QK-DBN, by utilizing only qualitative knowledge (QK) to make quantitative probabilistic inference. In the full Bayesian approach, we consider the model's uncertainty in probabilistic inference and perform probabilistic inference by model averaging: given evidence E, qualitative knowledge Ω and quantitative observation D, the (averaged) conditional distribution of the remaining variable X is calculated by integrating over the models:
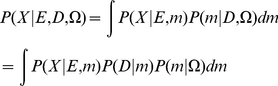
(1)where P(D|m) is the likelihood of the model and P(m|Ω) represents the model's prior probability given the qualitative knowledge. In the extreme case, there is no available quantitative data, i.e. D = null. It is still possible to make Bayesian probabilistic inference of Eq. 1 based on the knowledge Ω alone and the evidence E.
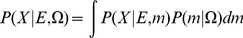
(2)Each DBN model *m* is determined by its structure and parameter vector. The Bayesian model space (all possible DBN models) is thus defined by: 1) a set of model structures **S** = {s_k_, k = 1,…,K}; 2) for each structure s_k_, a continuous ensemble of conditional probability table (CPT) configurations 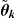. The BN/DBN model space can be written as **M** = {(s_k_, 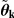),k = 1,…,K}. For every structure s_k_, each possible parameterization in the CPT configuration ensemble 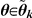 defines a member BN/DBN i.e. m = {(s_k_,**θ**)|k = 1,…,K} and the distribution of a single BN/DBN model is normalized against all models as
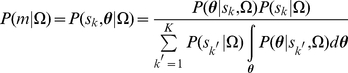
(3)where 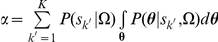 is the normalization scalar.
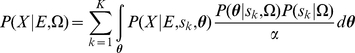
(4)We assume that the qualitative knowledge Ω regarding the network structure is consistent and certain, i.e. expert is fully certain about the dependence and direction of the influential relationships between two variables. Then the probability distribution of the model structure P(s_k_|Ω) is a Dirac delta function peaked at a specified structure s_k_, 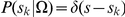. Given the k-th model structure, the qualitative constraints define a set of possible parameter configurations 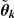 (see Text S2). Thusly, the conditional probability of each parameter vector **θ** given the k-th structure and qualitative constraints 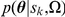 is equal to the probability of this vector belonging to the set of possible parameter configurations 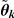 defined by the constraints in Ω, i.e. 
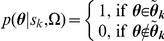
.

Therefore, the normalization factor 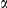 in Eq. 3 and 4 is equal to the size of the constrained parameter space 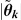.
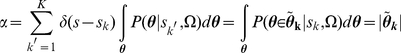
(5)Combining Eq. 1 to 5, we get:
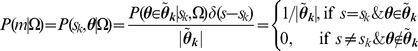
(6)

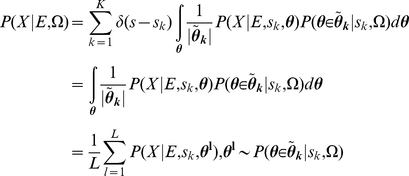
(7)Integration during Bayesian inference (in Eq. 4) can become intractable by analytical methods. In this case, we employ Markov chain Monte Carlo (MCMC) to compute the empirical value of the inference in Eq. 7. To efficiently generate samples satisfying the constraints, we exploited a rejection sampling method. The idea is to generate more samples from the current “unexplored” region so that the entire parameter space can be explored evenly. First, we generated samples from the proposed distribution and then rejected the samples inconsistent with constraints. The second step was to enhance sampling in the under-sampled space (see Text S2 for details).

### Inference in QK-DBN

We built a DBN by unrolling the cyclic hES network (Figure 1B). Since outgoing interfaces separate the current network from the past, only the potential function over the outgoing interface is required when forwarding the network belief at the current step to the next step. Computationally, we need to store this vector of information. The size of this vector is 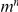 where *n* is the number of the variables in the outgoing interface and *m* is the number of the discrete values a variable can take (*m* = 2 in this study). To reduce computational cost and memory load, we should keep *n* as small as possible. We have developed a scheme to identify an optimal set of interface nodes. We firstly employed depth-first search [51] to identify all the nodes involved in the non-repeating loops in the curated genetic network as candidates for the interface nodes (Table S1 in Text S2), which are presumably important for the network's stochastic dynamics. The candidate interface nodes involved in all the non-repeating loops are listed in Table S2 and Figure S3 in Text S2. Next, to reduce inference complexity, we minimized the interface set. In particular, we ranked all candidates by a heuristic score = *B/(1−A)*. If this candidate is a must-cut node (loops must be cut at this node in order to keep the unrolled graph acyclic, such as auto-regulation node), *A = 1*, then its score becomes positive infinity (maximum). Otherwise *A = 0* and score = *B*. *B* is the number of total loops of which this node is a member. If a node is not in a loop, then *B = 0* and score = *0*. The values of A and B associated with each interface candidate are listed in Table S2 in Text S2. We iteratively picked nodes with the biggest score value from the list and cut all outgoing edges which are part of any loop from this node. We repeated this step until all loops were broken. All selected nodes compose the outgoing interface and these nodes are {NANOG, SP1, Oct4-Sox2, CDX2, PIAS1, GATA6, FOXA2, FOXA1} (yellow-colored nodes in Figure 1B).

Once these interface nodes were identified, we used the interface algorithm [52] to convert the DBN into junction tree and performed the message-passing algorithm [53] in the junction tree to infer both the joint probability over all variables and the marginal probability of each variable. After message-passing converged and the junction tree became a consistent tree [53], we calculated the joint probability over all variables 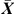 in the junction tree as 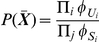, where 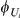 and 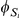 represent the cluster and sepset potentials respectively. The marginal probability of a variable ***X*** was calculated by 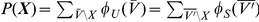, i.e. we could pick any cluster **U** or sepset **S** that contains the variable X and integrate out its potential function against other variables in this cluster or sepset.

### Predicting gene expression changes in human ES cells

Let **G** = {g_1_, g_2_,…,g_N_}, represent the gene expression levels of the genes in the network. We assumed the nodes in the DBN model are binary variables which take value of 0 or 1. Value “0” means that this gene is minimally expressed and “1” means is maximally expressed. The probability of a gene being max-/min-expressed (under condition E) is a continuous value in the range of [0,1]. When a gene is max-expressed, the probability of its node being “1” is 1, i.e. P(g_i_ = 1|E) = 1. When a gene is min-expressed, the probability of its node being “1” is 0, i.e. P(g_i_ = 1) = 0. Therefore, we consider this probability positively proportional to the expression level. The higher the probability of g_i_ = 1 is, the higher the gene's expression level is.

Let g_i,max_ and g_i,min_ represent the maximum and minimum expression level of the i-th gene g_i_, respectively. g_i_|E is the expression level of g_i_ under condition E and 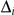 is g_i_ expression range. The (marginal) probability/belief of g_i_ being max-/min-expressed is a random value in [0,1] which is linearly proportional to the expression level (intensity) of this node:
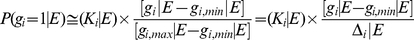
(8)where K_i_ is a constant. We can further simplify the above equation by rescaling the minimum expression level of g_i_ to 0 and the expression range to [0, g_i,max_|E]. In this case, the probabilities can be simplified as:
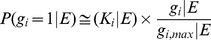
(9)The gene expression ratios between two conditions can be directly evaluated as:
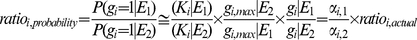
(10)where 
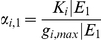
 and 
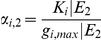
 are unknown scalars. The ratio between the probabilities is linearly proportional to the ratio between the gene expression levels. E_1_ and E_2_ are two experimental conditions, such as a control and a knockdown experiment, which are modeled as evidence in DBN. Therefore, we can predict the ratios of the gene expressions between knockdown and control experiments by calculating the ratios between the marginal probabilities of this gene under these conditions.

### Energy landscape in the cell state space

In this study, we assumed that cell states can be uniquely defined by the expression levels of all genes in the genetic network. We can calculate the potential energy of each state as 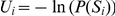, where P(S_i_) is the probability of i-th state of the network and U_i_ is the potential energy of this state. A collection of the potential energy values of all states in this network can be represented as 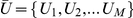, where M = 2^N^ and N is the number of genes in the constructed network. We can calculate all potential energy values in 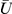 from the converged junction tree (see above). To compute the landscape of the genetic network, we need to consider the potentials under all possible (at least most representative) conditions. For our purpose of studying iPSC generation and the differentiation of the hESC, we chose to mimic the most representative scenarios during iPSC generation by varying the expression levels of the three master regulators OCT4, SOX2 and NANOG in hESCs from 0 to 1 with a small interval of 0.2. In DBN inference, for each combination of the levels of these regulators, we clamped their probabilities accordingly and simulated 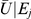 (Energy under j-th condition). Lastly, we calculated and normalized 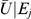 for all possible j, and then sum them to get the full landscape.

### Searching for recipes to generate iPSC

Let E_1_ denote the hESC state. Since the three master hESC regulators OCT4, SOX2 and NANOG are max-expressed in hESC, without losing generality, we clamped their marginal probability to 1 in our simulation. Then, by QK-DBN inference, we calculated the marginal probabilities of all the genes in the network in the hESC and these probabilities formed a vector of probabilities 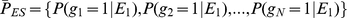. Similarly, let E_2_ represent the perturbation conditions specified by an iPS recipe. To search for iPS recipes in our simulation, starting from the differentiation states (OCT4, SOX2 and NANOG initialized to 0), we evolved the DBN given a specific reprogramming perturbation. Consequently, by QK-DBN, for each reprogramming recipe E_2_, we calculated the marginal probabilities for all the genes in the network given this perturbation. These marginal probabilities under E_2_ also form a vector 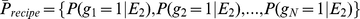. Since these marginal probabilities are proportional to their gene expression levels, we could directly evaluate a recipe by comparing vectors 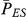 and 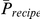. We employed root-mean-square distance (RMSD), Pearson correlation, and Spearman correlation to evaluate the distance from 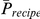 to 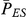.

### Depicting pathways of iPSC generation

We explored the cell state transition pathways during reprogramming. As mentioned above, the cell state is defined by the expression levels of all the genes in the network. In DBN, 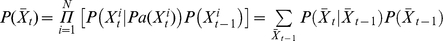, where 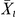 and 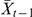 denote the expression levels of all genes at time t and t−1 respectively. We formulated the probability propagation in DBN for cell states as 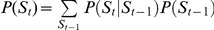, where 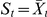 and 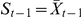 denote the cell state at time t and time (t−1). The probability of the current cell state is equal to the integration of the product of state transition probability 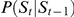 and the cell state probability at the last time step 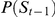. To simplify the computation, we applied maximum-a-posterior (MAP) estimation to predict the state-transition pathway. Namely, at each time step t, we picked the state which maximizes the cell state posterior at the current time step as the current cell state, 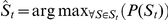. Note that the estimated pathway by MAP is not necessarily global maximum.

## Supporting Information

Dataset S1 **Possible reprogramming recipes with overexpression only.**
(XLSX)

Dataset S2 **Possible reprogramming recipes with overexpression and knockdown.**
(XLSX)

Text S1 **Supplementary Results.**
(PDF)

Text S2 **Supplementary Methods.**
(PDF)
