# Supplementary material for: Systematic Search for Recipes to Generate Induced Pluripotent Stem Cells
Source: PLoS Comput Biol. 2011 Dec 22;7(12):e1002300. doi: 10.1371/journal.pcbi.1002300 (PMC3245295; doi:10.1371/journal.pcbi.1002300)
Supplement: Text S1 — Supplementary Results. (PDF) [file pcbi.1002300.s003.pdf]

Supplementary Materials: Supplementary Results

Table S1. Proteins included in the curated genetic network.

AFP	bCAT*	BMP2	BMP2K	cAMP*	CDX2	CDX4	CEBP
**CSH1**	E2F4	FOXA1	FOXA2	FOXD3	FOXO1A	GATA2	GATA3
**GATA4**	GATA6	GDF3	hCGa*	hCGb*	KLF4	LEF1	LEF1-bCat
**LMCD1**	Mad	Mad-Max	MAX	MYC	Myc-Max	MYC-SP1	NANOG
**NFYA**	OCT4	Oct4-FOXD3	Oct4-Sox2	PBX1	PIAS1	PIASy	PRDM14
**SALL4**	SOX17	SOX2	SP1	SP3	SUMO1	T	TCF3
**TDGF1**	ZFP42*	ZIC3	ZNF206*				

*bCAT= Beta-catenin; CTNNB1; cAMP=cyclic-AMP; hCGa=CGA; hCGb=CGB; ZFP42=REX1; ZNF206=ZSCAN10;

Table S2. Edges and supporting evidence in the curated genetic network (Edge Number: 124; Activation: 85; Repression: 39)

Parent Node	Child Node	Interaction Type (1=activation; 0=repression)	References
**FOXA1**	AFP	1	(1, 2)
**FOXA2**	AFP	1	(3)
**SOX2**	BMP2	0	(4),(5),(6)
**NANOG**	BMP2	0	(7),(8)
**BMP2K**	BMP2	1	(9)
**HNF4A**	BMP2K	1	(10)
**OCT4**	CDX2	0	(7),(11)
**NANOG**	CDX2	0	(7),(12)
**CDX2**	CDX2	1	11, 67
**SP1**	CEBP	0	(13),(14)
**E2F4**	CEBP	0	(7),(15),(16)
**PRDM14**	CSH1	0	(17)
**FOXD3**	FOXA1	1	(18)
**OCT4-FOXD3**	FOXA1	0	(18),(19)
**FOXA1**	FOXA1	1	(18)
**FOXD3**	FOXA2	1	(18)
**OCT4-FOXD3**	FOXA2	0	(18),(19)
**FOXA2**	FOXA2	1	(18)
**SOX17**	FOXA2	1	(20),(21),(22),(23),(24),(18),(25),(3)
**NANOG**	FOXD3	1	(7),(26)
**OCT4**	FOXD3	1	(18),(27),(7)
**SOX2**	FOXD3	1	(7),(26)
**E2F4**	FOXD3	1	(7),(28),(29)
**OCT4**	FOXO1A	1	(7),(26)
**SOX2**	FOXO1A	1	(7),(26)
**NANOG**	FOXO1A	1	(7),(26)

**SUMO1**	GATA2	0	(30)
**PIASy**	GATA2	0	(30)
**SOX2**	GATA2	0	(31)
**NANOG**	GATA2	0	(31)
**NANOG**	GATA3	0	(7),(32)
**OCT4**	GATA4	0	(7),(11),(33)
**SUMO1**	GATA4	1	(34)
**PIAS1**	GATA4	1	(34)
**NANOG**	GATA4	0	(7),(11),(33)
**PRDM14**	GATA6	0	(17)
**NANOG**	GATA6	0	(7),(35),(12),(36)
**OCT4**	GATA6	0	(7),(27)
**GATA4**	GATA6	0	(37)
**LMCD1**	GATA6	0	(38)
**GATA6**	GATA6	1	(39-42)
**NANOG**	GDF3	1	(7),(43)
**OCT4**	hCGa	0	(32),(44),(33),(45),(46)
**GATA2**	hCGa	1	(47)
**GATA3**	hCGa	1	(47)
**cAMP**	hCGa	1	(48),(49),(50)
**OCT4**	hCGb	0	(32),(44),(33),(45),(46)
**SP1**	hCGb	1	(51)
**cAMP**	hCGb	1	(48),(49),(50)
**SP3**	hCGb	1	(52),(53)
**GATA6**	HNF4A	1	(54, 55)
**bCAT**	HNF4A	1	(56)
**OCT4**	KLF4	1	(7),(57)
**SOX2**	KLF4	1	(7),(57)
**NANOG**	KLF4	1	(7),(57)
**NANOG**	LEF1	0	(7),(58)
**OCT4**	LEF1	0	(7),(57)
**LEF1**	LEF1-bCat	1	(59)
**bCAT**	LEF1-bCat	1	(59)
**NANOG**	LMCD1	1	(7),(58)
**cAMP**	LMCD1	0	(60)
**MAX**	Mad-Max	1	(61, 62)
**Mad**	Mad-Max	1	(61, 62)
**PRDM14**	MYC	1	(63)
**E2F4**	MYC	1	(7),(15),(16)
**LEF1-bCat**	MYC	1	(64)
**TCF3**	MYC	0	(65, 66)
**MYC**	Myc-Max	1	(67)
**MAX**	Myc-Max	1	(67)

**MYC**	MYC-SP1	1	(68)
**SP1**	MYC-SP1	1	(68)
**ZIC3**	NANOG	1	(69)
**NANOG**	NANOG	1	(7)
**Oct4-Sox2**	NANOG	1	(7)
**PBX1**	NANOG	1	(70)
**KLF4**	NANOG	1	(70)
**GATA6**	NANOG	0	(39-42)
**SOX2**	NFYA	1	(7),(71)
**E2F4**	NFYA	1	(7),(15),(16)
**NANOG**	OCT4	1	(7)
**Oct4-Sox2**	OCT4	1	(7)
**CDX2**	OCT4	0	(11, 72)
**OCT4**	OCT4-FOXD3	1	(18)
**FOXD3**	OCT4-FOXD3	1	(18)
**OCT4**	Oct4-Sox2	1	(7)
**SOX2**	Oct4-Sox2	1	(7)
**NANOG**	PBX1	1	(7),(70)
**PIAS1**	PIAS1	1	(73)
**E2F4**	PIAS1	1	(7),(74)
**PIAS1**	PIASy	1	(73)
**OCT4**	PRDM14	1	(7, 75),(57)
**SOX2**	PRDM14	1	(7, 75),(57)
**NANOG**	PRDM14	1	(7, 75),(57)
**LEF1**	SALL4	1	(76)
**NANOG**	SALL4	1	(77),(26)
**SOX2**	SALL4	1	(77),(26)
**ZIC3**	SOX17	0	(69)
**OCT4**	SOX17	0	4,(78, 79)
**NANOG**	SOX2	1	4
**Oct4-Sox2**	SOX2	1	4
**E2F4**	SP1	1	4,(80)
**SP1**	SP1	1	(80)
**SP3**	SP1	0	(80)
**CEBP**	SP1	1	(80)
**NFYA**	SP1	1	(80)
**MYC-SP1**	SP1	0	(68)
**SUMO1**	SP3	0	(81, 82)
**PIAS1**	SP3	0	(81, 82)
**NANOG**	SUMO1	1	4
**E2F4**	SUMO1	1	4,(83),(28)

**NANOG**	T	0	4,(12, 84)
**PRDM14**	T	0	(17)
**E2F4**	T	0	4,(74)
**OCT4**	T	0	4,(11)
**E2F4**	TCF3	1	4
**OCT4**	TDGF1	1	(11)
**NANOG**	TDGF1	1	4,(17)
**SOX2**	TDGF1	1	4,(57)
**NANOG**	ZFP42	1	(7, 85)
**OCT4**	ZFP42	1	(7, 11)
**SOX2**	ZIC3	1	4,(26)
**NANOG**	ZIC3	1	(7, 69)
**NANOG**	ZNF206	1	4,(57)

Table S3. 22 Marker genes for hESCs and differentiated cells.

**Pluripotency Markers**	**OCT4, SOX2,NANOG,Oct4-Sox2,KLF4,FOXD3,ZIC3,ZFP42,GDF3, TDGF1,PBX1**
**Differentiation Markers**	FOXA2, AFP, SOX17, GATA4, GATA6, T, GATA2, GATA3, hCGa*, hCGb*, CDX2

*hCGa=CGA; hCGb=CGB

Figure S1a-f. Pearson correlation coefficient (r) and p-value (p) between predicted and experimental gene expression changes (in log2). (a)-(c): Day 3, 5 and 7 after knocking down OCT4 by Won et al.(77); (d)-(e) Day 2 and 3 after knocking down OCT4 by Kunarso et al.(86); (f) Day 4 after knocking down NANOG by Hyslop et al.(12).

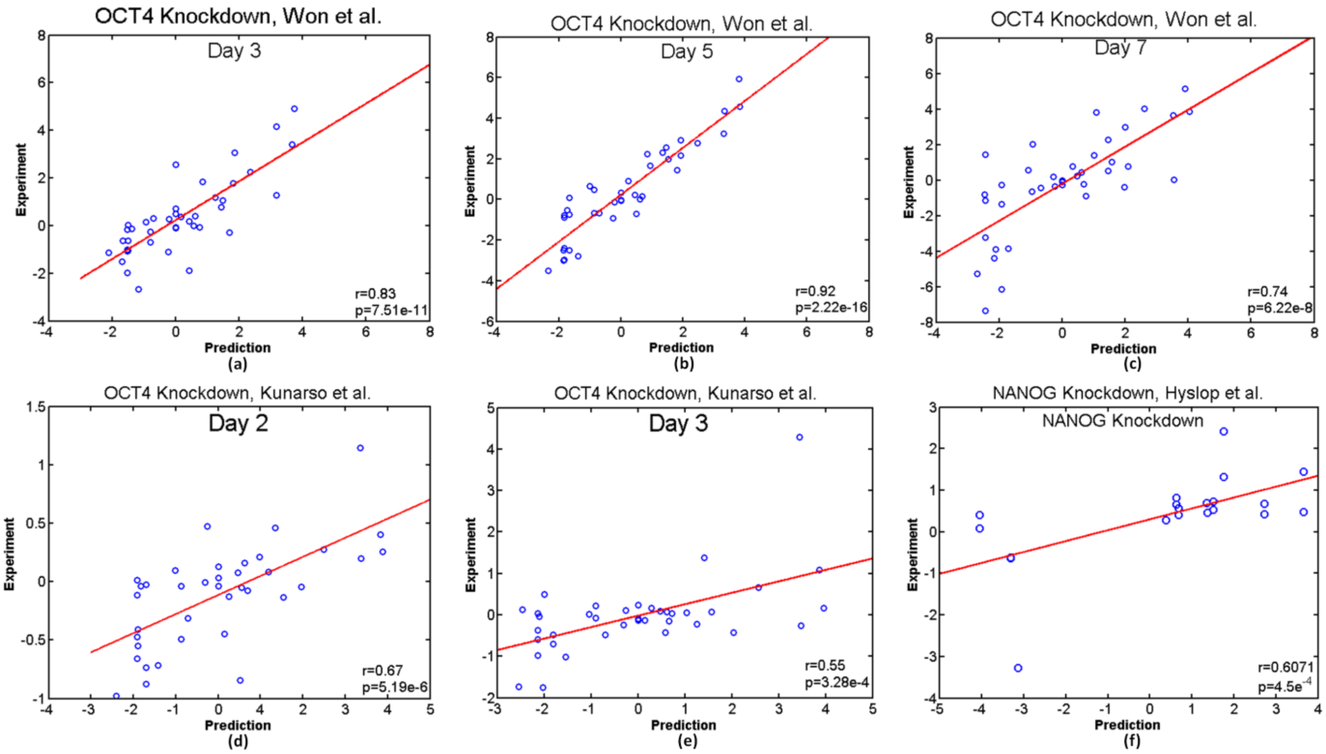

Figure S2. Reprogramming efficiency and gene expression similarity. The gene expression similarity is represented by RMSD, Pearson correlation, and Spearman rank correlation.

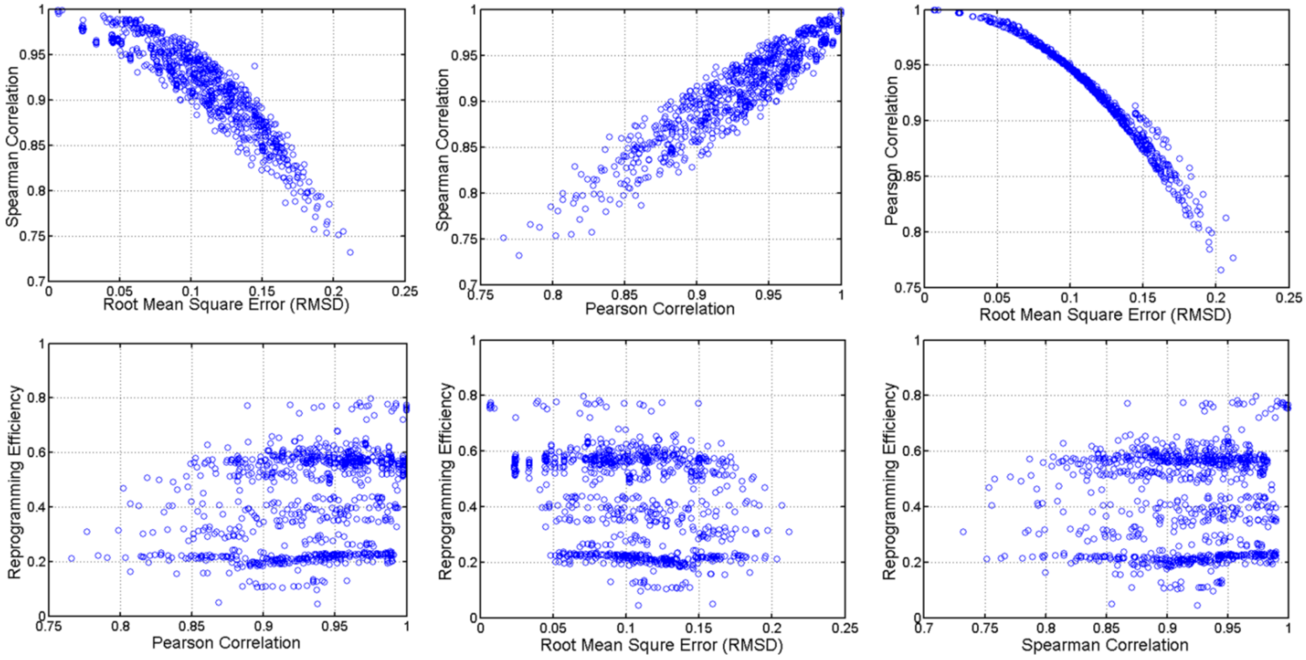

Figure S3. Distribution of Reprogramming Recipe's RMSD, Pearson and Spearman correlation.

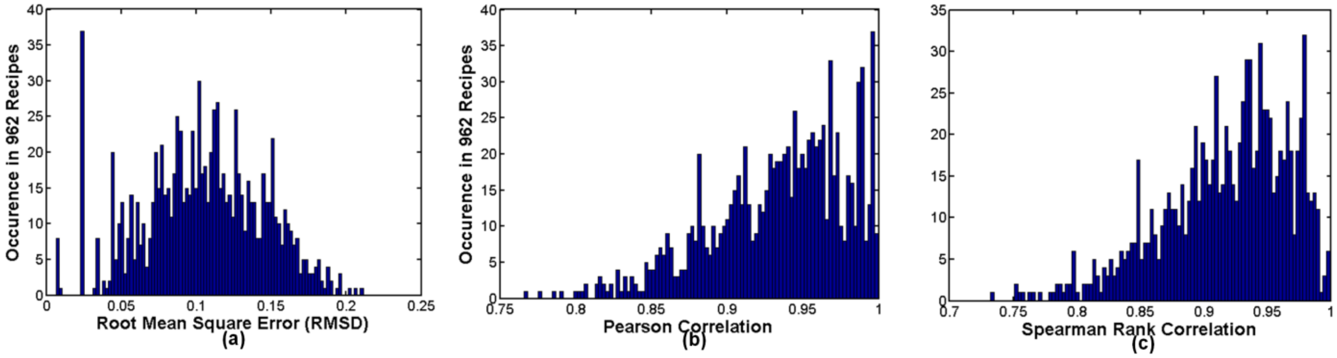

Figure S4. Reprogramming efficiencies of experimental recipes plus single gene knockdown. The experimental recipes are shown at the right end. The additional knockdown genes are shown in the order of reprogramming efficiency. If a component of a recipe is also knocked down, knockdown overwrites overexpression in the simulation.

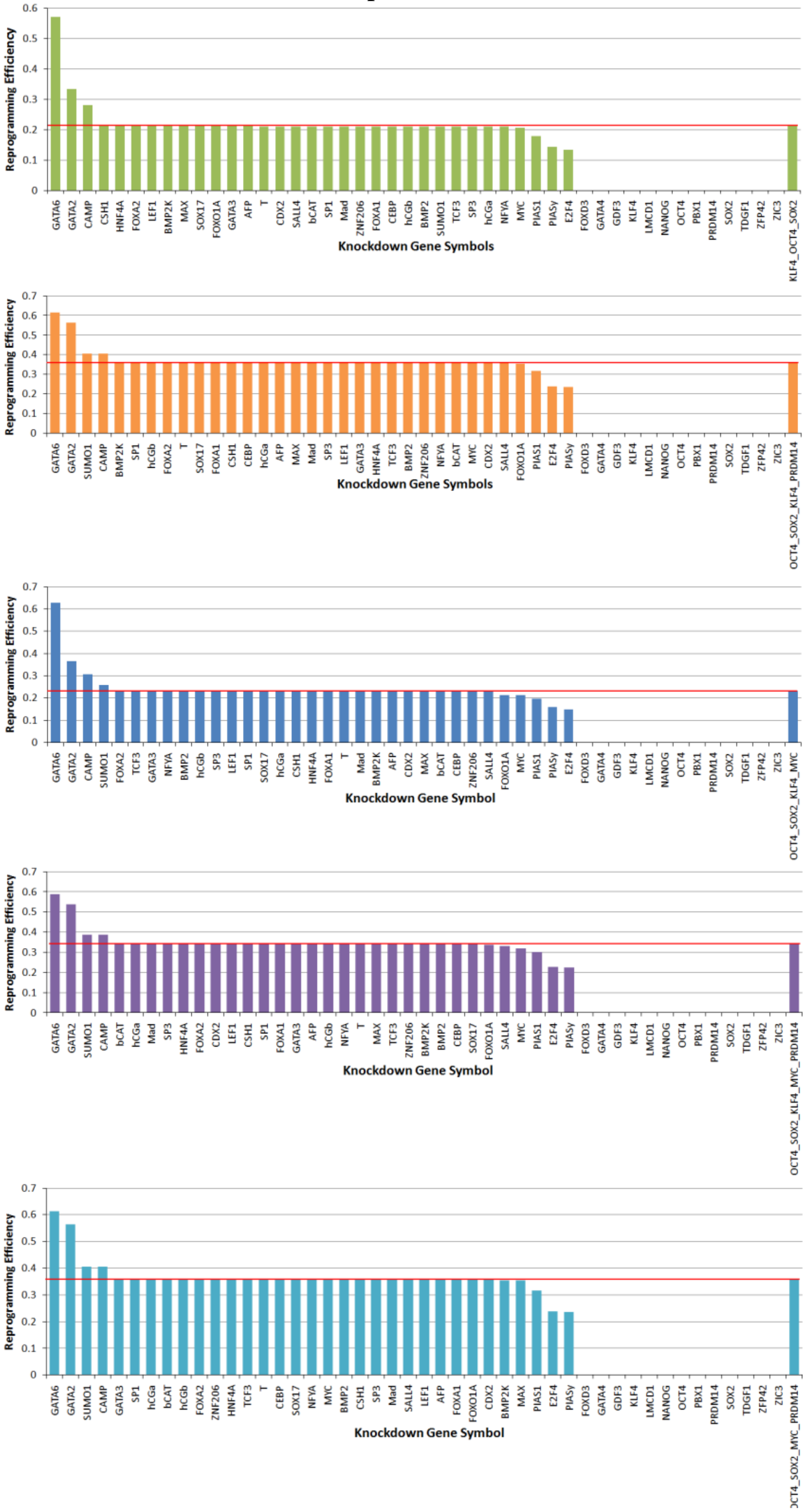

References:

1. Alison J. Crowe LS, Kelly Ke Li, Kathleen C. Lee, Brett T. Spear and Michelle C. Barton (1999) *The Journal of Biological Chemistry* **274**.
2. Kathleen C. Lee AJC, and Michelle Craig Barton (1999) *Molecular Cell Biology* **19,** 1279-1288.
3. Peterson R, Clevidence D, Ye H, & Costa R (1997) *Cell Growth Differ* **8,** 69-82.
4. Dhara SK & Stice SL (2008) *Journal of Cellular Biochemistry* **105,** 633-640.
5. Chew J-L, Loh Y-H, Zhang W, Chen X, Tam W-L, Yeap L-S, Li P, Ang Y-S, Lim B, Robson P*, et al.* (2005) *Mol. Cell. Biol.* **25,** 6031-6046.
6. Bajpai R, Coppola G, Kaul M, Talantova M, Cimadamore F, Nilbratt M, Geschwind DH, Lipton SA, & Terskikh AV (2009) *Cell Death Differ* **16,** 807-825.
7. Boyer LA, Lee TI, Cole MF, Johnstone SE, Levine SS, Zucker JP, Guenther MG, Kumar RM, Murray HL, Jenner RG*, et al.* (2005) *Cell* **122,** 947-956.
8. Darr H, Mayshar Y, & Benvenisty N (2006) *Development* **133,** 1193-1201.
9. EntrezGene.
10. D'Alessandro A, Righetti PG, & Zolla L (2009) *Journal of Proteome Research* **9,** 144-163.
11. Yasmin Babaie RH, Boris Greber, Thore C. Brink, Wasco Wruck, Detlef Groth, Hans Lehrach, Tom Burdon, James Adjaye (2007) *Stem Cells* **25,** 500-510.
12. Hyslop L, Stojkovic M, Armstrong L, Walter T, Stojkovic P, Przyborski S, Herbert M, Murdoch A, Strachan T, & Lako M (2005) *Stem Cells* **23,** 1035-1043.
13. Timchenko N, Wilson D, Taylor L, Abdelsayed S, Wilde M, Sawadogo M, & Darlington G (1995) *Mol. Cell. Biol.* **15,** 1192-1202.
14. Qi-Qun Tang M-SJ, and M. Daniel Lane (1999) *Molecular and Cellular Biology* **19,** 4855-4865.
15. Paul R. van Ginkel K-MH, Hilde Schjerven and Peggy J. Farnham *The Journal of Biological Chemistry* **272,** 18367-18374.
16. Weinmann AS, Bartley SM, Zhang T, Zhang MQ, & Farnham PJ (2001) *Mol. Cell. Biol.* **21,** 6820-6832.
17. Tsuneyoshi N, Sumi T, Onda H, Nojima H, Nakatsuji N, & Suemori H (2008) *Biochemical and Biophysical Research Communications* **367,** 899-905.
18. Guo Y, Costa R, Ramsey H, Starnes T, Vance G, Robertson K, Kelley M, Reinbold R, Scholer H, & Hromas R (2002) *Proceedings of the National Academy of Sciences of the United States of America* **99,** 3663-3667.
19. Rodriguez RT, Velkey JM, Lutzko C, Seerke R, Kohn DB, O'Shea KS, & Firpo MT (2007) *Exp. Biol. Med.* **232,** 1368-1380.
20. D'Amour KA, Agulnick AD, Eliazer S, Kelly OG, Kroon E, & Baetge EE (2005) *Nat Biotech* **23,** 1534-1541.
21. Yao S, Chen S, Clark J, Hao E, Beattie GM, Hayek A, & Ding S (2006) *Proceedings of the National Academy of Sciences* **103,** 6907-6912.
22. Sinner D, Rankin S, Lee M, & Zorn AM (2004) *Development* **131,** 3069-3080.
23. Palasingam P, Jauch R, Ng CKL, & Kolatkar PR (2009) *Journal of Molecular Biology* **388,** 619-630.
24. Odom DT, Dowell RD, Jacobsen ES, Nekludova L, Rolfe PA, Danford TW, Gifford DK, Fraenkel E, Bell GI, & Young RA (2006) *Mol Syst Biol* **2**.
25. Pani L, Quian XB, Clevidence D, & Costa RH (1992) *Mol. Cell. Biol.* **12,** 552-562.
26. Fong H, Hohenstein KA, & Donovan PJ (2008) *Stem Cells* **26,** 1931-1938.
27. Lukas Chavez ASB, Martin Vingron, Hans Lehrach, James Adjaye, Ralf Herwig (2009) *BMC Genomics* **10**.
28. Weinmann AS, Yan PS, Oberley MJ, Huang TH-M, & Farnham PJ (2002) *Genes & Development* **16,** 235-244.
29. Alkhateeb A, Fain PR, & Spritz RA (2005) *J Investig Dermatol* **125,** 388-391.

30. Chun T-H, Itoh H, Subramanian L, Iniguez-Lluhi JA, & Nakao K (2003) *Circ Res* **92,** 1201-1208.
31. Shi-Jiang Lu JAH, Qiang Feng, Jason D Hipp, Robert Lanza and Anthony Atala (2007) *Genome Biology* **8**.
32. Schulz LC, Ezashi T, Das P, Westfall SD, Livingston KA, & Roberts RM (2008) *Placenta* **29,** 10-16.
33. Hay DC, Sutherland L, Clark J, & Burdon T (2004) *Stem Cells* **22,** 225-235.
34. Jun Wang X-hFaRJS (2004) *The Journal of Biological Chemistry* **279,** 49091-49098.
35. Yasuda S-y, Tsuneyoshi N, Sumi T, Hasegawa K, Tada T, Nakatsuji N, & Suemori H (2006) *Genes to Cells* **11,** 1115-1123.
36. Singh AM, Hamazaki T, Hankowski KE, & Terada N (2007) *Stem Cells* **25,** 2534-2542.
37. Charron F, Paradis P, Bronchain O, Nemer G, & Nemer M (1999) *Mol. Cell. Biol.* **19,** 4355-4365.
38. Rath N, Wang Z, Lu MM, & Morrisey EE (2005) *Mol. Cell. Biol.* **25,** 8864-8873.
39. Rossant J & Tam PPL (2009) *Development* **136,** 701-713.
40. Spruce T, Pernaute B, Di-Gregorio A, Cobb BS, Merkenschlager M, Manzanares M, & Rodriguez TA (2010) *Developmental cell* **19,** 207-219.
41. Chazaud C, Yamanaka Y, Pawson T, & Rossant J (2006) *Developmental cell* **10,** 615-624.
42. Niwa H (2007) *Development* **134,** 635-646.
43. Clark AT, Rodriguez RT, Bodnar MS, Abeyta MJ, Cedars MI, Turek PJ, Firpo MT, & Reijo Pera RA (2004) *Stem Cells* **22,** 169-179.
44. Matin MM, Walsh JR, Gokhale PJ, Draper JS, Bahrami AR, Morton I, Moore HD, & Andrews PW (2004) *Stem Cells* **22,** 659-668.
45. Liu L, Leaman D, Villalta M, & Roberts RM (1997) *Mol Endocrinol* **11,** 1651-1658.
46. Roberts LLaRM (1996) *The Journal of Biological Chemistry* **271,** 16683-16689.
47. Steger DJ, Hecht JH, & Mellon PL (1994) *Mol. Cell. Biol.* **14,** 5592-5602.
48. Wade Johnson CA, Stuart Handwerger, Trevor Williams, Richard G. Pestell, J. Larry Jameson (1997) *The Journal of Biological Chemistry* **272,** 15405-15412.
49. JAMESON JL & HOLLENBERG AN (1993) *Endocr Rev* **14,** 203-221.
50. Ringler GE, Lee-Chuan K, Miller WL, & Strauss Iii JF (1989) *Molecular and Cellular Endocrinology* **61,** 13-21.
51. Jameson WJaJL (1999) *Molecular Endocrinology* **13,** 1963-1975.
52. Rull K & Laan M (2005) *Human Reproduction* **20,** 3360-3368.
53. Knofler M, Saleh L, Bauer S, Galos B, Rotheneder H, Husslein P, & Helmer H (2004) *Endocrinology* **145,** 1685-1694.
54. Morrisey EE, Tang Z, Sigrist K, Lu MM, Jiang F, Ip HS, & Parmacek MS (1998) *Genes & Development* **12,** 3579-3590.
55. Shureiqi I, Zuo X, Broaddus R, Wu Y, Guan B, Morris JS, & Lippman SM (2007) *The FASEB Journal* **21,** 743-753.
56. Monga GZaSS (2009) *The FASEB Journal* **23**.
57. Greber B, Lehrach H, & Adjaye J (2007) *BMC Developmental Biology* **7,** 46.
58. Sperger JM, Chen X, Draper JS, Antosiewicz JE, Chon CH, Jones SB, Brooks JD, Andrews PW, Brown PO, & Thomson JA (2003) *Proceedings of the National Academy of Sciences of the United States of America* **100,** 13350-13355.
59. Behrens J, von Kries JP, Kuhl M, Bruhn L, Wedlich D, Grosschedl R, & Birchmeier W (1996) *Nature* **382,** 638-642.
60. Peter Dudek DP (2008) *PLoS ONE* **26**.
61. Ayer DE, Kretzner L, & Eisenman RN (1993) *Cell* **72,** 211-222.
62. Zervos AS, Gyuris J, & Brent R (1993) *Cell* **72,** 223-232.
63. Nishikawa N, Toyota M, Suzuki H, Honma T, Fujikane T, Ohmura T, Nishidate T, Ohe-Toyota M, Maruyama R, Sonoda T*, et al.* (2007) *Cancer Research* **67,** 9649-9657.

64. Sebastien Taurin NS, Yimin Qin, Darren Browning and Nickolai O. Dulin (2006) *The Journal of Biological Chemistry* **281,** 9971-9976.
65. Cole MF, Johnstone SE, Newman JJ, Kagey MH, & Young RA (2008) *Genes & Development* **22,** 746-755.
66. Spieker N, Peterson J, Reneman S, & Destrée O (2004) *Developmental Dynamics* **231,** 510-517.
67. Blackwood EM, Lüscher B, Kretzner L, & Eisenman RN (1991) *Cold Spring Harbor Symposia on Quantitative Biology* **56,** 109-117.
68. Gartel AL & Shchors K (2003) *Experimental Cell Research* **283,** 17-21.
69. Lim LS, Loh Y-H, Zhang W, Li Y, Chen X, Wang Y, Bakre M, Ng H-H, & Stanton LW (2007) *Mol. Biol. Cell* **18,** 1348-1358.
70. Chan KK-K, Zhang J, Chia N-Y, Chan Y-S, Sim HS, Tan KS, Oh SK-W, Ng H-H, & Choo AB-H (2009) *Stem Cells* **27,** 2114-2125.
71. Marica Grskovic CC, Alexandre Gaspar-Maia, Hao Li, and Miguel Ramalho-Santos (2007) *PLoS Genetics* **3**.
72. Niwa H, Toyooka Y, Shimosato D, Strumpf D, Takahashi K, Yagi R, & Rossant J (2005) *Cell* **123,** 917-929.
73. Jiann-An Tan SHH, Katherine G. Hamil, Gail Grossman, Peter Petrusz and Frank S. French (2002) *The Journal of Biological Chemistry* **277,** 16993-17001.
74. Becker KA, Stein JL, Lian JB, van Wijnen AJ, & Stein GS (2007) *Journal of Cellular Physiology* **210,** 517-526.
75. Dettman EJ & Justice MJ (2008) *PLoS ONE* **3,** e3823.
76. Böhm J, Sustmann C, Wilhelm C, & Kohlhase J (2006) *Biochemical and Biophysical Research Communications* **348,** 898-907.
77. Won KJea (2010) *Submitted*.
78. Stefanovic S, Abboud N, Désilets S, Nury D, Cowan C, & Pucéat M (2009) *The Journal of Cell Biology* **186,** 665-673.
79. Desbordes SC, Placantonakis DG, Ciro A, Socci ND, Lee G, Djaballah H, & Studer L (2008) *Cell stem cell* **2,** 602-612.
80. Marta Nicolás VN, and Carlos J Ciudad (2003) *Biochemical Journal* **408**.
81. Alexandra Sapetschnig FK, Grigore Rischitor, Trientje Mennenga and Guntram Suske (2004) *The Journal of Biological Chemistry* **279,** 42095-42105.
82. Sapetschnig A, Rischitor G, Braun H, Doll A, Schergaut M, Melchior F, & Suske G (2002) *EMBO J* **21,** 5206-5215.
83. Nanos-Webb A, Deyrieux A, Bian X-l, Rosas-Acosta G, & Wilson V (2010) *Molecular Biology Reports* **37,** 1155-1163.
84. Pankratz MT, Li X-J, LaVaute TM, Lyons EA, Chen X, & Zhang S-C (2007) *Stem Cells* **25,** 1511-1520.
85. Initiative TISC (2007) *Nat Biotech* **25,** 803-816.
86. Kunarso G, Chia N-Y, Jeyakani J, Hwang C, Lu X, Chan Y-S, Ng H-H, & Bourque G (2010) *Nat Genet* **42,** 631-634.
