# Supplementary material for: Systematic Search for Recipes to Generate Induced Pluripotent Stem Cells
Source: PLoS Comput Biol. 2011 Dec 22;7(12):e1002300. doi: 10.1371/journal.pcbi.1002300 (PMC3245295; doi:10.1371/journal.pcbi.1002300)
Supplement: Text S2 — Supplementary Methods. (PDF) [file pcbi.1002300.s004.pdf]

Supplementary Materials: Supplementary Methods

Table of Contents

1. Modeling genetic network using Dynamic Bayesian network (DBN)
    1.1 Modeling Cyclic Network using DBN
        1.1.1 Unrolling Cyclic Network
        1.1.2 Identifying interface nodes
    1.2 Convert Knowledge to Constraints and Parameter Estimation
        1.2.1 Preliminary
        1.2.2 Constraint-based Qualitative Knowledge Model
        1.2.3 Parameter Constraints for Genetic Network

2 Novel Qualitative Knowledge-based (Dynamic) Bayesian Network (QK-DBN) Inference
    2.1 Preliminary and Notations
    2.2 Qualitative Knowledge-based (Dynamic) Bayesian network Inference (QK-BN, QK-DBN)
    2.3 Efficient Monte Carlo Markov Chain
    2.4 Probabilistic Inference in Dynamic Bayesian Network
        2.4.1 Interface Algorithm
        2.4.2 Belief propagation and Message-passing

3. Apply QK-DBN to the curated genetic network
    3.1Gene Expression Changes Prediction in Human ES Cells
    3.2 Energy Landscape in the cell state space
    3.3 Searching for recipes to generate iPSC
    3.4 Depicting pathways of iPSC generation

1. Modeling genetic network using Dynamic Bayesian network (DBN)

To search for reprogramming recipes, we need to make *de novo* predictions of the phenotypic consequences of perturbations to a genetic network. We use expression levels of all the genes in the network to represent phenotypes, i.e. ES or differentiation state. We choose dynamic Bayesian network (DBN)(1) to model the curated hESC network (Figure 1) that contain many feedback loops. Prediction of consequences of a perturbation is an inference problem in DBN. Genomic data, either perturbation (gene knockdown or overexpression) or temporal gene expression data, that are conventionally used to train the parameters or learn the structure of the DBN(1), are very limited in hESC. Therefore, we take a new method(2, 3) based on the constraint imposed by the network structure on the parameter space to conduct inference. We showed that this method achieved a satisfactory performance on predicting gene expression changes upon perturbation in the hESC (Figure 2 and SR1). Our model also allows efficient calculation of the potential landscape in the cell state space and monitoring the reprogramming progress in this landscape.

We first describe how to build the DBN by unrolling the cyclic genetic network. We next lay out the framework of learning constraints of the parameters in the DBN model from the network topology and literature knowledge and how to implement this framework to model the genetic network. Then, we show how to conduct inference in the constraint-based DBN model and how to predict gene expression changes in the genetic network using this method. Finally, we illustrate the application of our method to calculate the potential landscape in the cell state space.

1.1 Modeling Cyclic Network using DBN

1.1.1 Unrolling Cyclic Network

The curated genetic network in hESCs contains many cyclic regulations (Figure 1). In this section, we discuss in detail how to transform a cyclic network into a dynamic Bayesian network (DBN), which is designed to capture the dynamics of a cyclic network by decomposing the feedback loops into a series of temporal structures.

Definition 1.1.1 A cyclic genetic network is denoted by G. A DBN is defined by its initial static model $\mathbf{B_0}$ and dynamic model $\mathbf{B_t}$, i.e. ($\mathbf{B_0}$, $\mathbf{B_t}$). The dynamic process of G with a DBN model can be described as:

$$(\mathbf{B_0}, \mathbf{B_t}) = f(G) \quad (1)$$

where f denotes the graphical transformation from G to ($\mathbf{B_0}$, $\mathbf{B_t}$).

The cyclic network of a biological system (G) does not indicate how to determine the set of interface nodes for unrolling. Thusly, we need to assume that the temporal dynamics of the cyclic biological network are triggered by the feedback links and the nodes involved in these links are natural candidates for cutting the loops to unroll G.

Definition 1.1.2 An unrolling scheme $\mathbf{f_b}$ is defined as a transformation of G into a DBN. Since the DBN represents a process which is stationary and Markovian, a DBN can be alternatively depicted by a 2TBN consisted of the first two time slices of a process, i.e. $f_b(G)=(\mathbf{B_1}, \mathbf{B_2}, \mathbf{B_{12}})$, where $\mathbf{B_1}= \mathbf{B_2}=G(\mathbf{V \backslash V'})$ and $\mathbf{B_{12}=V'}$. $\mathbf{B_1}$ is the first time slice network and $\mathbf{B_2}$ is the second time

slice network. $\mathbf{B_{12}}$ represents the edges from the outgoing interface ($\mathbf{I_1}$) in B1 to their incoming nodes ($\mathbf{I_2}$) in B2. $\mathbf{B_1}$ and $\mathbf{B_2}$ are intra-slice models and $\mathbf{B_{12}}$ represents the transitional links from the first time slice $\mathbf{B_1}$ to the second time slice $\mathbf{B_2}$. **V** denotes all the edges in G. Let **X** denote the set of outgoing interface ($\mathbf{I_1}$) nodes in the first time slice $\mathbf{B_1}$ and **Y** the set of incoming interface ($\mathbf{I_2}$) nodes in the second-slice $\mathbf{B_2}$. **V'** denotes all the edges between **X** and **Y**. An example is shown in Figure S1.

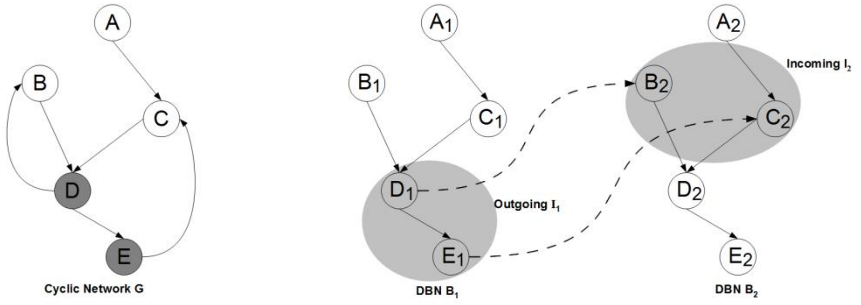

Figure S1. Unrolling cyclic network. The shaded nodes represent the cut nodes in G. **X** include D_1 and E_1. **Y** include B_2 and C_2.

There might be multiple strategies for unrolling the cycles in G if the interface nodes are unknown. In principle, any node in a loop can be used to break the loop. Multiple nodes may also be shared by two or more cycles which can be used to cut the loops in G. For example, in Figure S1, there are two loops: i) B->D->B and ii) C->D->E->C. For the first loop, there are two possible choices on the interface nodes, i.e. B or D. For the second loop, there are three possible choices, i.e. C, D and E. These different cutting patterns will result in different message-passing pathways and computational complexity. As shown in Figure S2, in the upper panel, D is the only interface node and the outgoing edges from D to B and from D to E are unrolled; in the lower panel, D and C are selected as interface nodes, the outgoing edges from D to B and from C to D are unrolled.

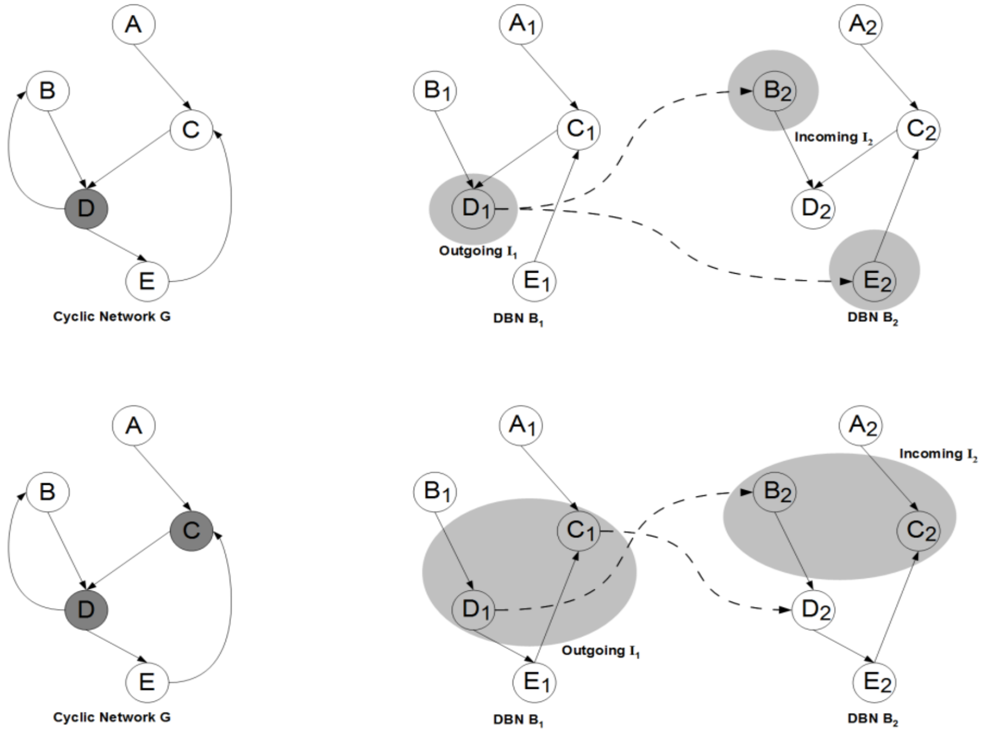

Figure S2. Examples of possible unrolling schemes

1.1.2 Identifying interface nodes

DBN represents a stochastic process with an initial model and a series of dynamic models whose stationary posterior probability distribution converge to its true value after sufficient steps. The system dynamics and delays between two consecutive time steps are invoked by the belief transition and updates between outgoing interface ($\mathbf{I_1}$) in the last time step and the incoming interface ($\mathbf{I_2}$) in the next time step of a DBN. Therefore, the outgoing and incoming interfaces of a DBN should contain nodes whose state transitions and belief changes trigger the whole network (system) evolvement. Since outgoing interfaces separate the current network from the past, only the potential function over the outgoing interface is required when forwarding the network belief at the current step to the next step. Computationally, we need to store this vector of information. The size of this vector is m^n where *n* is the number of the discrete-value variables in the outgoing interface and *m* is the number of the discrete values a variable can take. In this paper, we assume all nodes in the DBN are binary variables, i.e. *m*=2. To reduce computational cost and memory load, we should keep *n* as small as possible.

In the cyclic network, the stochastic dynamics of a system is dominated by the loopy links between the nodes. Therefore, we have developed a scheme to identify an optimal set of interface nodes to maintain the temporal properties of the original network G and reduce the computational complexity incurred by this unrolling scheme. We firstly employed depth-first search(4) to identify all the nodes involved in the non-repeating loops in the curated genetic network as candidates for the interface nodes (Table S1), which are presumably important for the network's stochastic dynamics. The candidate interface nodes involved in all the non-repeating loops are listed in Table S2 and Figure S3. Next, to reduce inference complexity, we minimize

the interface set. In particular, we rank all candidates by a heuristic score=$B/(1-A)$. If this candidate is a must-cut node (loops must be cut at this node in order to keep the unrolled graph acyclic, such as auto-regulation node), $A=1$, then its score becomes positive infinity (maximum). Otherwise $A=0$ and score=B. B is the number of total loops of which this node is a member. If a node is not in a loop, then $B=0$ and score=0. The values of A and B associated with each interface candidate is listed in Table S2. We iteratively pick nodes with the biggest score value from the list and cut all outgoing edges which are part of any loop from this node. We repeat this step until all loops are broken. All selected nodes compose the outgoing interface and these nodes are {NANOG, SP1, Oct4-Sox2, CDX2, PIAS1, GATA6, FOXA2, FOXA1} (yellow-colored nodes in Figure 1B in the main text).

Table S1. All non-repeating Loops in the curated genetic network

Loop	Number of genes
**NANOG -> ZIC3 -> SOX2 -> Oct4-Sox2 -> OCT4 -> NANOG**	5
**Oct4-Sox2 -> SOX2 -> NANOG -> GATA6 -> OCT4 -> Oct4-Sox2**	5
**Oct4-Sox2 -> SOX2 -> NANOG -> KLF4 -> OCT4 -> Oct4-Sox2**	5
**Oct4-Sox2 -> SOX2 -> NANOG -> NANOG -> GATA6 -> OCT4 -> Oct4-Sox2**	5
**Oct4-Sox2 -> SOX2 -> NANOG -> NANOG -> KLF4 -> OCT4 -> Oct4-Sox2**	5
**NANOG -> KLF4 -> OCT4 ->CDX2 -> NANOG**	4
**GATA6 -> GATA4 -> NANOG -> GATA6**	3
**GATA6 ->LMCD1 -> NANOG -> GATA6**	3
**GATA6 -> OCT4 ->NANOG -> GATA6**	3
**GATA6 -> PRDM14 ->NANOG -> GATA6**	3
**NANOG -> KLF4 -> OCT4 -> NANOG**	3
**NANOG -> KLF4 -> SOX2 -> NANOG**	3
**NANOG -> Oct4-Sox2 -> OCT4 -> NANOG**	3
**NANOG -> Oct4-Sox2 -> SOX2 -> NANOG**	3
**NANOG -> ZIC3 -> SOX2 -> NANOG**	3
**Oct4-Sox2 -> SOX2 -> NANOG -> NANOG -> Oct4-Sox2**	3
**Oct4-Sox2 -> SOX2 -> NANOG -> Oct4-Sox2**	3
**CDX2 ->OCT4 -> CDX2**	2
**CEBP ->SP1 -> CEBP**	2
**GATA6 -> NANOG -> GATA6**	2
**MYC-SP1 -> SP1 -> MYC-SP1**	2
**NANOG -> KLF4 -> NANOG**	2
**NANOG -> ZIC3 -> NANOG**	2
**OCT4 -> Oct4-Sox2 -> OCT4**	2
**Oct4-Sox2 -> SOX2 -> Oct4-Sox2**	2
**PBX1 -> NANOG -> PBX1**	2
**CDX2 -> CDX2**	1
**FOXA1 -> FOXA1**	1
**FOXA2 -> FOXA2**	1

**GATA6 -> GATA6**	1
**NANOG -> NANOG**	1
**SP1 -> SP1**	1
**PIAS1 -> PIAS1**	1

Table S2. All candidates of interface nodes in the curated genetic network

Genes	# of loops (B)*	Must-cut (A)*
**Oct4-Sox2**	11	0
**SOX2**	11	0
**NANOG**	22	0
**KLF4**	6	0
**OCT4**	11	0
**ZIC3**	3	0
**PBX1**	1	0
**FOXA2**	1	1
**FOXA1**	1	1
**PIAS1**	1	1
**SP1**	3	1
**CEBP**	1	0
**MYC-SP1**	1	0
**GATA6**	8	1
**CDX2**	3	1

# of loops=The number of loops that pass through a node. This value equals the B value. Must-cut indicates whether a node must be cut to unroll a cycle, e.g. auto-regulation: 1=true and 0=false. This value is equal to the A value.

Figure S3. Number of loops for each node in the curated genetic network

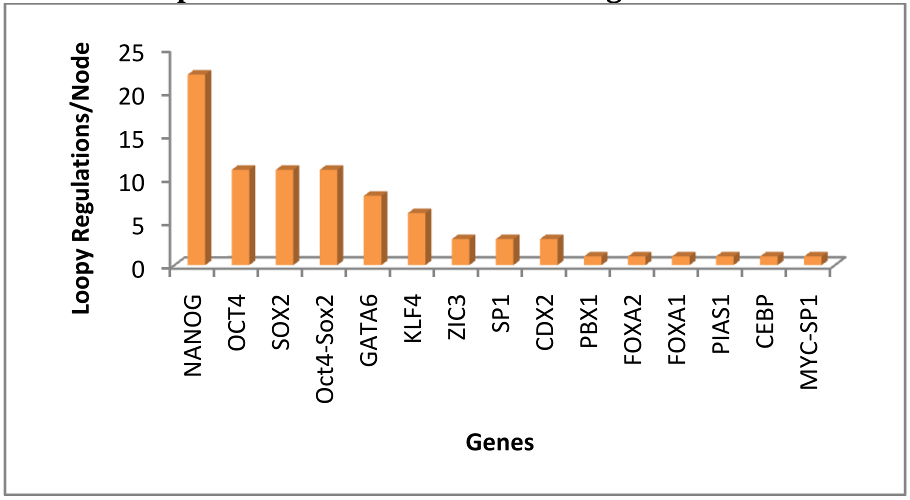

1.1.3 Interface Algorithm

In this section, we briefly describe the interface algorithm using an example. The interface algorithm(1) begins with constructing two junction trees for $\mathbf{B_0}$ and $\mathbf{B_t}$ in a DBN model (see definition 1.1.1) respectively. For smoothing, filtering and predicting, we only need to query those values based on the distribution over the interface nodes at the current time step. To do this, we use the clique potential of the outgoing interface ($\mathbf{I_1}$) from the previous time step to encapsulate all required information of the process up to the present time step. In the context of junction trees, this means maintaining the potential of the clique which contains the interface at each time step. Thus, we need to ensure that the outgoing interface is fully contained within at least one clique of each junction tree. After the junction trees for $\mathbf{B_0}$ and $\mathbf{B_t}$ are constructed respectively, standard junction tree inference is performed to compress all the beliefs and information into the interface clique potential. This resulted potential of interface clique enters the same junction tree (same structure) at the next time step. This iterative process continues until the Markovian chain (chain of junction trees) is converged.

We demonstrate how the interface algorithm works on a simple example in Figure S4 to S6 and summarize the algorithm in Table S3. The junction tree for the curated genetic network is shown in Figure S7.

In the first step (Figure S4), we create a junction tree J_0 from B_0. Given a 2TBN B_t, we recover B_0 by removing all the nodes in the second time slice of B_t ($B_{t,2}$) and all the edges emitted from the 1st time slice of B_t ($B_{t,1}$). Next, B_0 is moralized, producing a moral graph. Then, in order to ensure that the outgoing interface I_0 is fully contained within at least one clique of each junction tree, edges are added between all nodes in I_0. Finally, we employ standard junction tree triangulation, formation, and clique potential initialization process to produce the junction tree J_0. The clique containing the outgoing interface I_0 in J_0 is labeled as the in-/out-clique.(1, 5, 6)

In the second step (Figure S5), we firstly identify the interface nodes in the outgoing interface of time slice 1 and 2. We represent these nodes as I_1 and I_2. In general, we can use I_{t-1} and I_t to represent the interface at time slice 1 and 2 of any t-th 2TBN. Next, we create a “1.5-slice DBN” (H_t) from the 2TBN B_t by removing all non-interface nodes in the 1st time slice of B_t while keeping the interface nodes I_1 (or I_{t-1}), Next, we construct a junction tree, J_t, for each H_t. In order to keep track of the joint probability of the interface nodes $P(I_{t-1})$ and $P(I_t)$, we must enforce the constraint that I_{t-1} and I_t each forms a clique. This can be ensured by simply adding edges to the moral graph between all nodes in I_{t-1}, and similarly for I_t, before constructing J_t. We can now glue all the junction trees together via their interfaces. Then we triangulate J_t the same way as we triangulated J_0. The junction tree is created as before, but clique potential initialization proceeds slightly differently than in the static case. When initializing clique potentials, only conditional probability tables (CPTs) of nodes in time slice 2 of the original 2TBN are multiplied onto cliques in the junction tree[1, 5].

The third step (Figure S6) is performed iteratively. Once the junction trees have been constructed and initialized, inference is performed through two stages of message-passing (see section 2.4). The clique containing the outgoing interface in slice t−1 is called the in-clique, while the clique containing the outgoing interface in slice t is called out-clique. At time t-1, the junction tree J_{t-1} is created and initialized for the (t-1)-th 1.5-slice DBN which consists of time

slices (t-1,t) and the inference is performed on J_{t-1} by message-passing. Once the forward-backward message passing is completed, the out-clique potential is marginalized down to the outgoing interface potential and the interface algorithm is ready to advance this inference to the next time step t. At time t, the junction tree J_t is constructed and initialized for the t-th 1.5-slice DBN which consists of time slices (t,t+1). The outgoing interface potential of J_{t-1} from last step is multiplied by the in-clique potential in the t-th 1.5-slice DBN. Note that since the 1.5-slice DBN structure is consistent, the j-tree structure of J_{t-1} is the same to that of J_t. Thusly, we can simply build the junction tree once and use it for all time steps at which inference is performed. At each time step, the junction tree only needs to be re-initialized to its initial clique potentials[1, 5].

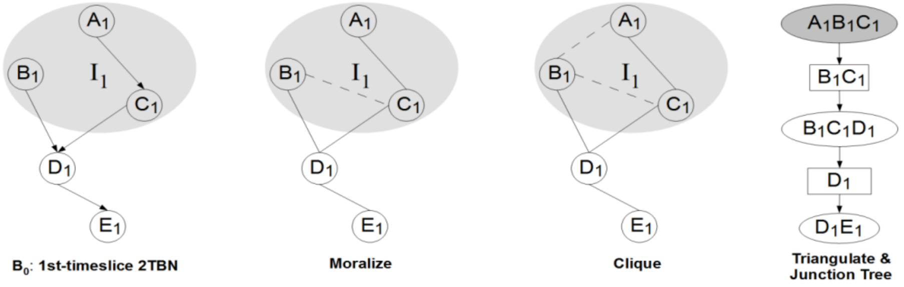

Figure S4. Step 1 of Interface Algorithm: Construct junction tree J_0 from 1st-time slice of 2TBN B_0

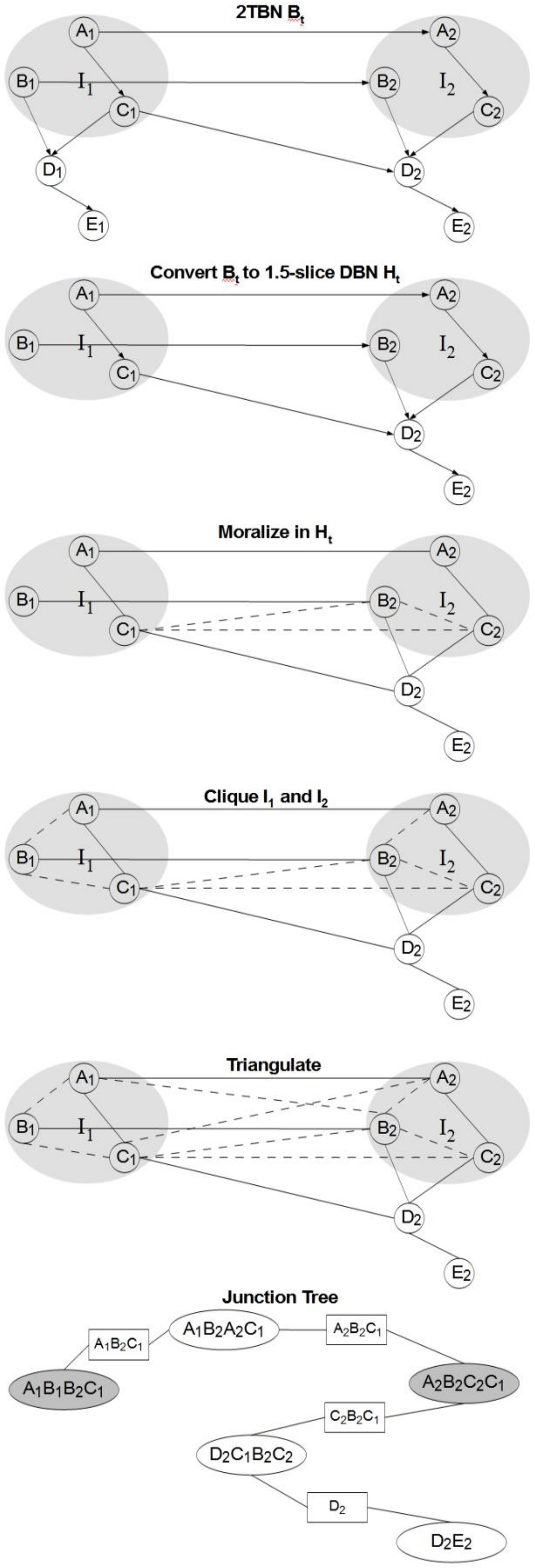

Figure S5. Step 2 of Interface Algorithm: Construct junction tree J_t from 1.5-slice of 2TBN H_t.

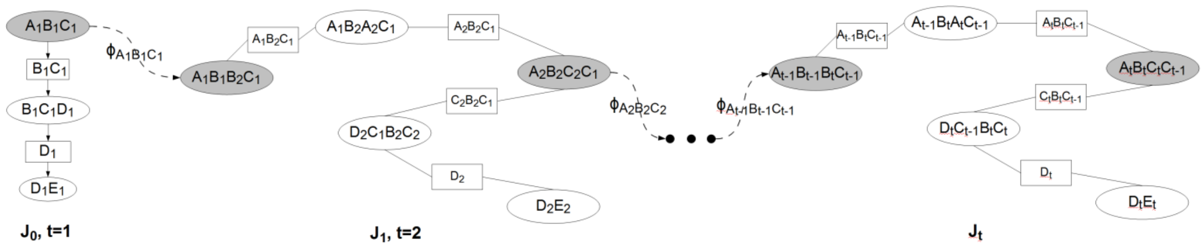

Figure S6. Step 3 of Interface Algorithm: Iterative "forward advance" inference through message-passing.

Table S3. Summary of Interface Algorithm

Step 1: Creation and Initialization of J_0	• Remove all the nodes in time slice 2 from a 2TBN<br>• Identify nodes in outgoing interface of time slice 1, name it I_1<br>• Moralize, add edges to make I_1 a clique<br>• Triangulate and find cliques for J_0<br>• Identify in- and out- cliques that contains I_1<br>• Initialize clique potentials to 1s and multiply nodes CPTs onto cliques (same to static BN)
Step 2: Creation and Initialization of J_t	• Begin with the whole 2TBN, identify nodes in interface of time slice 1 and slice 2 respectively, name them I_1 and I_2<br>• Convert 2TBN to 1.5-slice DBN by removing non-interface nodes in time slice 1<br>• Moralize and add edges to make I_1 and I_2 each a clique<br>• Triangulate and find cliques for J_t<br>• Identify in-clique that contains I_1 and out-clique that contains I_2<br>• Initialize clique potentials to 1s and only multiply time slice 2 nodes' CPTs from a clique onto that clique's potential
Step 3: Message-passing and Forward Advance	• Perform standard forward-backward message-passing at current time step and junction tree<br>• Marginalize the out-clique potential φ in the current junction tree down to the outgoing interface potential<br>• Increase time step and initialize the new junction tree J_{t+1}<br>• Multiply φ onto the in-clique potential of J_{t+1}

Figure S7. The junction tree of 2-TBN for curated network.

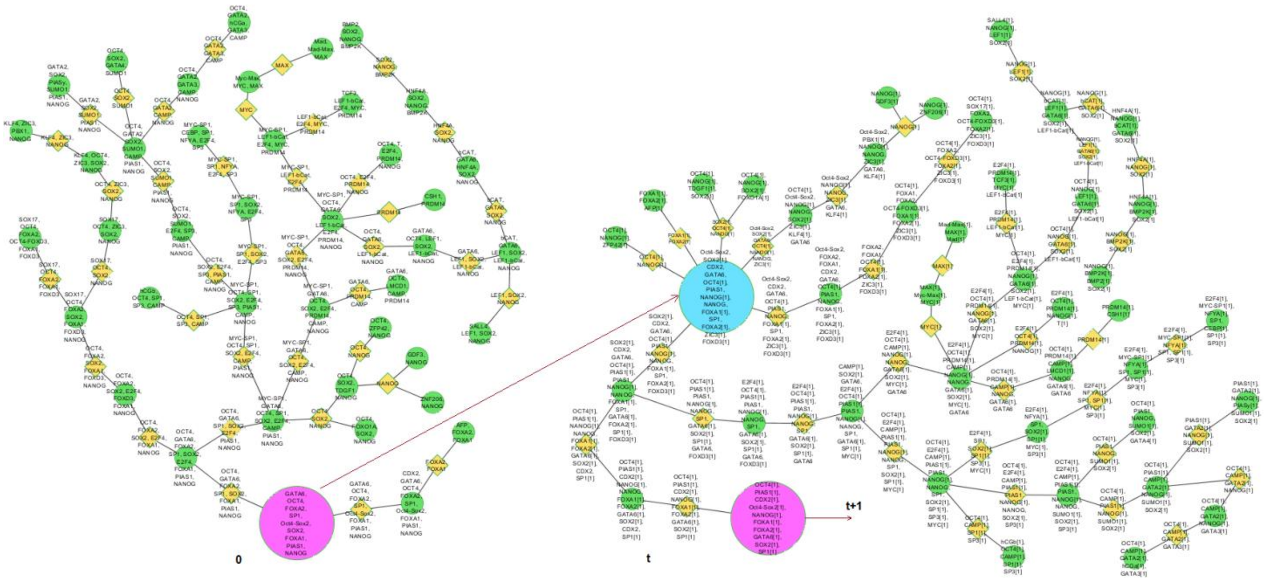

The green-colored nodes represent cliques and yellow nodes denote separate sets of nodes between contiguous cliques in the junction tree. The tree on the left represents the 0.5-TBN junction tree for t=0 and the tree on the right shows the 1.5-TBN junction tree for 2TBN at t>0. The outgoing interface, which contains the outgoing nodes in 0.5-TBN at t=0, is colored in red. The incoming and outgoing cliques in 1.5-TBN at t>0 are represented by pink- and blue-colored nodes, respectively. Belief messages from t=0 to t=1 are transmitted from the outgoing clique in 0.5-TBN (red) to the incoming clique in 1.5-TBN (blue). Belief messages for t>0 are passed through the outgoing clique (pink node) in the current 1.5-2TBN junction tree to the incoming clique (blue node) in the next time slice of the 1.5-TBN junction tree iteratively.

1.2 Convert Knowledge to Constraints and Parameter Estimation

After we use the interface algorithm to convert a DBN into junction tree, we need to parameterize the DBN before we can perform inference. There are many reverse-engineering approaches to estimate the parameters of a DBN model from quantitative data. However, the applications of these approaches are limited by data scarcity and high-dimensionality in modeling biological networks. In this section, we employ a model(2) to convert qualitative knowledge encoded in the network topology to a set of constraints in the DBN parameter space. These constraints are then used to recover the parameter distribution of the DBN model.

1.2.1 Preliminary

An edge in a DBN model encodes the conditional probability distribution (CPD) of a child node given a configuration of its parents. Each child node in the DBN model is associated with a CPD and all CPDs compile the vector of parameters in the DBN model which is denoted by $\boldsymbol{\theta}$. When we use the DBN model to represent a genetic network, each edge reflects a functional regulation, such as transcriptional regulation or protein-protein interaction. For example, assume a simple acyclic model with two nodes A and B. For the sake of simplicity, we restrict our discussion to binary-valued nodes. If the influence takes direction from A to B, A is the parent node and B is the child node. The CPD of B is the only parameter for the model, i.e.

$\boldsymbol{\theta}$={θ_B}. The CPD is a multinomial distribution and can be written in the form: θ_B ={P(B|A), P(B|$\bar{A}$), P($\bar{B}$|A), P($\bar{B}$|$\bar{A}$)}. Once the values of θ_B are known, it is straightforward to infer B's belief/probability given A's status.

Definition 1.2.1 *θ_{ijk} denotes the CPD entry of i-th node taking its k-th value given the j-th parents configuration: $\theta_{ijk} = P(X_i = k|\pi(X_i) = j)$.*

1.2.2 Constraint-based Qualitative Knowledge Model

Throughout this paper, we assume our nodes are binary variables. Logic "1" and "0" values of a node are defined as "present" and "absent" or "active" and "inactive" or "maximal expressed" and "minimally expressed" as synonyms, A and $\bar{A}$. Qualitative influences with directions can be defined based on the number of influences imposed from the parents on the child. There are three cases of influences(2), a single influence, a joint influence and a mixed joint influence. In addition, there are extra features added to these classes of influences(3). For the sake of clarity, we summarize these influences here.

1. Single Influence

Definition 1.2.2 *If a child node B has a parent node A and the parent imposes an isolated influence on the child, qualitative influence between parent and child is referred as single influence. Single influence can be further classified into single positive influence and single negative influence.*

Definition 1.2.3 *If presence of parent node A renders presence of child node B more likely, the parent node has a single positive influence on the child node. This can be represented by the inequality*

$$P(B|A) > P(B|\bar{A}) \quad (2)$$

Definition 1.2.4 *If presence of parent node A renders presence of child node B less likely, the parent node has a single negative influence on the child node. This can be represented by the inequality*

$$P(B|A) < P(B|\bar{A}) \quad (3)$$

2. Joint Influence

Definition 1.2.5 *If a child node B has more than one parent node and all parents influence the child in a joint way, these influences between parents and child is referred as joint influence. This joint influence can be either plain/independent, synergic (cooperative) or antagonistic (competitive) and the individual influences from the parents to the child can be either positive or negative.*

Definition 1.2.6 *If a joint influence from two or more parent nodes generates combined influential effects larger than the single effect from each individual parent and individual effects are independent to each other, the joint influence is referred as plain joint influence or independent joint influence.*

Definition 1.2.7 *If all individual influences from the parents to the child are positive, a plain joint is called positive plain joint or positive independent joint influence.*

$$P(C|A,B) > \begin{cases} P(C|A,\bar{B}) \\ P(C|\bar{A},B) \end{cases} > P(C|\bar{A},\bar{B}) \qquad (4)$$

Definition 1.2.8 *If all individual influences from the parents to the child are negative, a plain joint is called* <u>*negative plain joint*</u> *or* <u>*negative independent joint influence*</u>*.*

$$P(\bar{C}|A,B) > \begin{cases} P(\bar{C}|A,\bar{B}) \\ P(\bar{C}|\bar{A},B) \end{cases} > P(\bar{C}|\bar{A},\bar{B}) \qquad (5)$$

Definition 1.2.9 *If joint influences from two or more parent nodes generate a combined influential effect larger than the sum of each single effect from individual parents, the joint influence is referred as* <u>*cooperative joint influence*</u>

Definition 1.2.10 *If all individual influences from the parents to the child are positive, a cooperative influence is called* <u>*positive cooperative joint influence*</u>*.*

$$P(C|A,B) > [P(C|A,\bar{B}) + P(C|\bar{A},B)] > \begin{cases} P(C|A,\bar{B}) \\ P(C|\bar{A},B) \end{cases} > P(C|\bar{A},\bar{B}) \quad (6)$$

Definition 1.2.11 *If all individual influences from the parents to the child are negative, a cooperative influence is called* <u>*negative cooperative joint influence*</u>*.*

$$P(\bar{C}|A,B) > [P(\bar{C}|A,\bar{B}) + P(\bar{C}|\bar{A},B)] > \begin{cases} P(\bar{C}|A,\bar{B}) \\ P(\bar{C}|\bar{A},B) \end{cases} > P(\bar{C}|\bar{A},\bar{B}) \quad (7)$$

3. Mixed Joint Influence

Definition 1.2.12 *If the joint effect on a child is formed by a mixture of positive and negative individual influences from its parents, the extraction of a probability model is not well defined in general. Hence, we adopt the following scheme: If there are mixed influences from several parent nodes to a child node, and no additional information is given, they are treated as independent and with equal influential strength. Assume that parent node A imposes positive single influence on child node C and parent node B imposes negative single influence on child node C, the joint influence can be represented by*

$P(C|A,B) > P(C|\bar{A},B)$; $P(C|A,\bar{B}) > P(C|\bar{A},\bar{B})$; $P(C|A,\bar{B}) > P(C|A,B)$; $P(C|\bar{A},\bar{B}) >$ $P(C|\bar{A},B)$ (8)

4. Extra influential features

Some extra features(3) are designed to further refine the above knowledge model and parameter distributions. These features define relative and absolute properties of the conditional probability distributions, which include: 1) the ratio; 2) the difference between any two or more probabilities; 3) the absolute boundary of any probability entry.

Definition 1.2.13 *These properties impose additional restrictions on the DBN model uncertainty so that a more accurate generalization can be achieved. They can be compactly encoded by a linear regression function $f(\theta_{ijk}) \leq c$ (c is a scalar) and θ_{ijk} is defined by Def.1.2.1. In the case that node B imposes single influence on node A, there are two probabilistic configurations. The linear constraints can then be written as*

$$P(B|A) >,< R \times P(B|\bar{A}) + \Delta;\ P(B|A), P(B|\bar{A}) \epsilon [Bd_{min}, Bd_{max}] \qquad (9)$$

where R is influence ratio, Δ is influence difference and Bd is the boundary.

1.2.3 Parameter Constraints for Genetic Network

We use these constraints to characterize biological interactions in genetic networks. Roughly, biological interactions can be classified as: i) transcriptional regulations including activation and repression and ii) protein-protein regulatory interactions including protein complex formation, activation, and inhibition by post-translational modification. In DBN, these regulatory interactions are parameterized by one of the above constraints to confine the model uncertainty in the parameter space, i.e. $\widetilde{\boldsymbol{\theta}}$ in $P(\boldsymbol{\theta} \in \widetilde{\boldsymbol{\theta}} | \boldsymbol{s}, \Omega)$ (Table S4). A genetic network often consists of a combination of interactions belonging to different classes in Table S4, which can be translated to a set of constraints accordingly.

Table S4. Parameter constrains in DBN imposed by biological interactions

Transcriptional activation<br>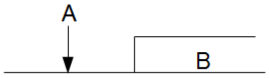 	Default: Single Positive Influence (Def.1.2.3)<br>$P(B\|A) > P(B\|\bar{A})$<br>Optional: Extra features (Def.2.2.13)<br>$P(B\|A) > R \times P(B\|\bar{A}) + \Delta$<br>$P(B\|A) \in [\alpha_{min}, \alpha_{max}]$;<br>$P(B\|\bar{A}) \in [\beta_{min}, \beta_{max}]$;
Transcriptional inhibition<br>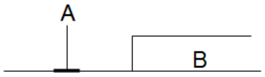 	Default: Single Negative Influence (Def.1.2.4)<br>$P(\bar{B}\|A) > P(\bar{B}\|\bar{A})$<br>Optional: Extra features (Def.2.2.13)<br>$P(\bar{B}\|A) > R \times P(\bar{B}\|\bar{A}) + \Delta$<br>$P(\bar{B}\|A) \in [\alpha_{min}, \alpha_{max}]$;<br>$P(\bar{B}\|\bar{A}) \in [\beta_{min}, \beta_{max}]$;
Multiple Individual Trans-activation<br>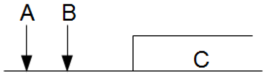 	Default: Positive Plain/Independent Joint (Def.1.2.7)<br>$P(C\|A,B) > \begin{cases} P(C\|A,\bar{B}) \\ P(C\|\bar{A},B) \end{cases} > P(C\|\bar{A},\bar{B})$<br>Optional: Extra features (Def.2.2.13)<br>$P(C\|A,B) > \begin{cases} R_1 \times P(C\|A,\bar{B}) + \Delta_1 \\ R_2 \times P(C\|\bar{A},B) + \Delta_2 \end{cases} > R_3 \times P(C\|\bar{A},\bar{B}) + \Delta_3$<br>$P(C\|A,B) \in [\alpha_{min}, \alpha_{max}]$;<br>$P(C\|A,\bar{B}) \in [\rho_{min}, \rho_{max}]$;<br>$P(C\|\bar{A},B) \in [\gamma_{min}, \gamma_{max}]$;<br>$P(C\|\bar{A},\bar{B}) \in [\beta_{min}, \beta_{max}]$;
Multiple Individual Trans-inhibition<br>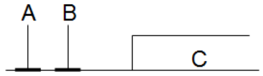 	Default: Negative Plain/Independence Joint (Def.1.2.8)<br>$P(\bar{C}\|A,B) > \begin{cases} P(\bar{C}\|A,\bar{B}) \\ P(\bar{C}\|\bar{A},B) \end{cases} > P(\bar{C}\|\bar{A},\bar{B})$<br>Optional: Extra features (Def.2.2.13)<br>$P(\bar{C}\|A,B) > \begin{cases} R_1 \times P(\bar{C}\|A,\bar{B}) + \Delta_1 \\ R_2 \times P(\bar{C}\|\bar{A},B) + \Delta_2 \end{cases} > R_3 \times P(\bar{C}\|\bar{A},\bar{B}) + \Delta_3$

<table>
<tr>
<td></td>
<td>$P(\bar{C}|A,B) \in [\alpha_{min}, \alpha_{max}]$;
$P(\bar{C}|A,\bar{B}) \in [\rho_{min}, \rho_{max}]$;
$P(\bar{C}|\bar{A},B) \in [\gamma_{min}, \gamma_{max}]$;
$P(\bar{C}|\bar{A},\bar{B}) \in [\beta_{min}, \beta_{max}]$;</td>
</tr>
<tr>
<td>Cooperative Trans-activation
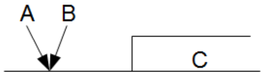
</td>
<td>Default: Positive Synergy (Def.1.2.10)
$P(C|A,B) > [P(C|A,\bar{B}) + P(C|\bar{A},B)] > \begin{cases} P(C|A,\bar{B}) \\ P(C|\bar{A},B) \end{cases} > P(C|\bar{A},\bar{B})$

Optional: Extra features (Def.2.2.13)
$P(C|A,B) > R_4 \times [P(C|A,\bar{B}) + P(C|\bar{A},B)] + \Delta_4 > \begin{cases} R_1 \times P(C|A,\bar{B}) + \Delta_1 \\ R_2 \times P(C|\bar{A},B) + \Delta_2 \end{cases} > R_3 \times P(C|\bar{A},\bar{B}) + \Delta_3$

$P(C|A,B) \in [\alpha_{min}, \alpha_{max}]$;
$P(C|A,\bar{B}) \in [\rho_{min}, \rho_{max}]$;
$P(C|\bar{A},B) \in [\gamma_{min}, \gamma_{max}]$;
$P(C|\bar{A},\bar{B}) \in [\beta_{min}, \beta_{max}]$.</td>
</tr>
<tr>
<td>Cooperative Trans-inhibition
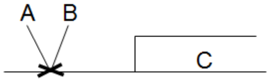
</td>
<td>Default: Negative Synergy (Def.1.2.11)
$P(\bar{C}|A,B) > [P(\bar{C}|A,\bar{B}) + P(\bar{C}|\bar{A},B)] > \begin{cases} P(\bar{C}|A,\bar{B}) \\ P(\bar{C}|\bar{A},B) \end{cases} > P(\bar{C}|\bar{A},\bar{B})$

Optional: Extra features (Def.2.2.13)
$P(\bar{C}|A,B) > R_4 \times [P(\bar{C}|A,\bar{B}) + P(\bar{C}|\bar{A},B)] + \Delta_4 > \begin{cases} R_1 \times P(\bar{C}|A,\bar{B}) + \Delta_1 \\ R_2 \times P(\bar{C}|\bar{A},B) + \Delta_2 \end{cases} > R_3 \times P(\bar{C}|\bar{A},\bar{B}) + \Delta_3$

$P(\bar{C}|A,B) \in [\alpha_{min}, \alpha_{max}]$;
$P(\bar{C}|A,\bar{B}) \in [\rho_{min}, \rho_{max}]$;
$P(\bar{C}|\bar{A},B) \in [\gamma_{min}, \gamma_{max}]$;
$P(\bar{C}|\bar{A},\bar{B}) \in [\beta_{min}, \beta_{max}]$.</td>
</tr>
<tr>
<td>Mixed inputs
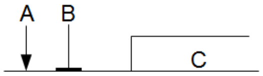
</td>
<td>Default: Mixed Joint Influence (Def1.2.12)
$P(C|A,B) > P(C|\bar{A},B)$; $P(C|A,\bar{B}) > P(C|\bar{A},\bar{B})$;
$P(C|A,\bar{B}) > P(C|A,B)$; $P(C|\bar{A},\bar{B}) > P(C|\bar{A},B)$;

Optional: Extra features (Def.2.2.13)
$P(C|A,B) > R_1 \times P(C|\bar{A},B) + \Delta_1$;
$P(C|A,\bar{B}) > R_2 \times P(C|\bar{A},\bar{B}) + \Delta_2$;
$P(C|A,\bar{B}) > R_3 \times P(C|A,B) + \Delta_3$;
$P(C|\bar{A},\bar{B}) > R_4 \times P(C|\bar{A},B) + \Delta_4$;

$P(C|A,B) \in [\alpha_{min}, \alpha_{max}]$;
$P(C|A,\bar{B}) \in [\rho_{min}, \rho_{max}]$;</td>
</tr>
</table>

	$P(C \mid \bar{A},B) \in [\gamma_{min},\gamma_{max}]$;<br>$P(C \mid \bar{A},\bar{B}) \in [\beta_{min},\beta_{max}]$.
Protein complex formation<br>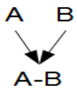	Default: Positive Plain/Independent Joint (Def.1.2.7)<br>$P(AB \mid A,B) > \begin{cases} P(AB \mid A,\bar{B}) \\ P(AB \mid \bar{A},B) \end{cases} > P(AB \mid \bar{A},\bar{B})$<br><br>Optional: Extra features (Def.2.2.13)<br>$P(AB \mid A,B) > \begin{cases} R_1 \times P(AB \mid A,\bar{B}) + \Delta_1 \\ R_2 \times P(AB \mid \bar{A},B) + \Delta_2 \end{cases}$<br>$> R_3 \times P(AB \mid \bar{A},\bar{B}) + \Delta_3$<br><br>$P(AB \mid A,B) \in [\alpha_{min},\alpha_{max}]$;<br>$P(AB \mid A,\bar{B}) \in [\rho_{min},\rho_{max}]$;<br>$P(AB \mid \bar{A},B) \in [\gamma_{min},\gamma_{max}]$;<br>$P(AB \mid \bar{A},\bar{B}) \in [\beta_{min},\beta_{max}]$.<br>(AB= protein complex)
Activation by post-translational modification<br>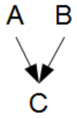	Default: Positive Synergy (Def.1.2.10)<br>$P(C \mid A,B) > [P(C \mid A,\bar{B}) + P(C \mid \bar{A},B)] > \begin{cases} P(C \mid A,\bar{B}) \\ P(C \mid \bar{A},B) \end{cases}$<br>$> P(C \mid \bar{A},\bar{B})$<br><br>Optional: Extra features (Def.2.2.13)<br>$P(C \mid A,B) > R_4 \times [P(C \mid A,\bar{B}) + P(C \mid \bar{A},B)] + \Delta_4$<br>$> \begin{cases} R_1 \times P(C \mid A,\bar{B}) + \Delta_1 \\ R_2 \times P(C \mid \bar{A},B) + \Delta_2 \end{cases} > R_3 \times P(C \mid \bar{A},\bar{B}) + \Delta_3$<br><br>$P(C \mid A,B) \in [\alpha_{min},\alpha_{max}]$;<br>$P(C \mid A,\bar{B}) \in [\rho_{min},\rho_{max}]$;<br>$P(C \mid \bar{A},B) \in [\gamma_{min},\gamma_{max}]$;<br>$P(C \mid \bar{A},\bar{B}) \in [\beta_{min},\beta_{max}]$.
Inhibition by post-translational modification<br>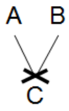	Default: Negative Synergy (Def.1.2.11)<br>$P(\bar{C} \mid A,B) > [P(\bar{C} \mid A,\bar{B}) + P(\bar{C} \mid \bar{A},B)] > \begin{cases} P(\bar{C} \mid A,\bar{B}) \\ P(\bar{C} \mid \bar{A},B) \end{cases}$<br>$> P(\bar{C} \mid \bar{A},\bar{B})$<br><br>Optional: Extra features (Def.2.2.13)<br>$P(\bar{C} \mid A,B) > R_4 \times [P(\bar{C} \mid A,\bar{B}) + P(\bar{C} \mid \bar{A},B)] + \Delta_4$<br>$> \begin{cases} R_1 \times P(\bar{C} \mid A,\bar{B}) + \Delta_1 \\ R_2 \times P(\bar{C} \mid \bar{A},B) + \Delta_2 \end{cases} > R_3 \times P(\bar{C} \mid \bar{A},\bar{B}) + \Delta_3$<br><br>$P(C \mid A,B) \in [\alpha_{min},\alpha_{max}]$;<br>$P(C \mid A,\bar{B}) \in [\rho_{min},\rho_{max}]$;<br>$P(C \mid \bar{A},B) \in [\gamma_{min},\gamma_{max}]$;

	$P(C\|\bar{A},\bar{B}) \in [\beta_{min}, \beta_{max}]$.

In this study of the hESC network, we use the default constraint in Table S4 to set parameter constraints for each local structure in the network. If not specified by qualitative knowledge, we heuristically set: R=1, Δ=0, $\alpha_{min}=0.9$, $\alpha_{max}=1.0$, $\beta_{min}=0.0$, and $\beta_{max}=0.1$ wherever these optional features apply. We do this to maintain consistency and generality across whole network. The selected boundary values reflect the observation of gene expression that is turned on or off by a positive or negative regulation. We set other parameters with default values, i.e. $\rho_{min}=0$, $\gamma_{min}=0$ and $\rho_{max}=1.0$, $\gamma_{max}=1.0$, except for protein complex, $\rho_{min}=0$, $\gamma_{min}=0$ and $\rho_{max}=0$, $\gamma_{max}=0$. A list of parameter constraints used for all local structures in hESC network is shown in Table S5. A specific configuration according to the qualitative knowledge was set for the complete inhibition of CGA and CGB by Oct4.

Table S5. Qualitative Knowledge Constraints for Genetic Regulatory Network

Child Node	Parent Nodes	Regulation Type[a]	Constraint Type[b]
**ZNF206**	NANOG	1	SP
**SALL4**	LEF1,NANOG,SOX2	1,1,1	PPJ
**SOX2**	NANOG,Oct4-Sox2	1,1	PPJ
**Oct4-Sox2**	OCT4,SOX2	1,1	PPJ
**FOXO1A**	OCT4,SOX2,NANOG	1,1,1	PPJ
**ZIC3**	SOX2,NANOG	1,1	PPJ
**PRDM14**	OCT4,SOX2,NANOG	1,1,1	PPJ
**LEF1-bCat**	LEF1,bCAT	1,1	PPJ
**ZFP42**	NANOG,OCT4	1,1	PPJ
**BMP2**	SOX2,NANOG,BMP2K	0,0,1	MJ
**Myc-Max**	MYC,MAX	1,1	PPJ
**Mad-Max**	MAX,Mad	1,1	PPJ
**OCT4**	NANOG,Oct4-Sox2,CDX2	1,1,0	MJ
**LEF1**	NANOG,OCT4	0,0	NPJ
**FOXD3**	NANOG,OCT4,SOX2,E2F4	1,1,1,1	PPJ
**OCT4-FOXD3**	OCT4,FOXD3	1,1	PPJ
**SOX17**	ZIC3,OCT4	0,0	NPJ
**FOXA2**	FOXD3,OCT4-FOXD3,FOXA2,SOX17	1,0,1,1	MJ
**FOXA1**	FOXD3,OCT4-FOXD3,FOXA1	1,0,1	MJ
**AFP**	FOXA1,FOXA2	1,1	PPJ
**CDX2**	OCT4,NANOG,CDX2	0,0,1	MJ
**T**	NANOG,PRDM14,E2F4,OCT4	0,0,0,0	NPJ
**SUMO1**	NANOG,E2F4	1,1	PPJ

**PIAS1**	E2F4,PIAS1	1,1	PPJ
**GATA4**	OCT4,SUMO1,SOX2,NANOG,PIAS1	0,1,0,0,1	MJ
**GDF3**	NANOG	1	SP
**TCF3**	E2F4	1	SP
**KLF4**	OCT4,SOX2,NANOG	1,1,1	PPJ
**CSH1**	PRDM14	0	SN
**NANOG**	ZIC3,NANOG,Oct4-Sox2,PBX1,KLF4,GATA6	1,1,1,1,1,0	MJ
**LMCD1**	NANOG,CAMP	1,0	MJ
**GATA6**	PRDM14,NANOG,OCT4,LMCD1,GATA6	0,0,0,0,1	MJ
**MYC**	PRDM14,E2F4,LEF1-bCat,TCF3	1,1,1,0	MJ
**GATA2**	SUMO1,PIASy,SOX2,NANOG	0,0,0,0	NPJ
**GATA3**	NANOG	0	SN
**hCGa**	OCT4,GATA2,GATA3,CAMP	0,1,1,1	MJ
**hCGb**	OCT4,SP1,CAMP,SP3	0,1,1,1	MJ
**SP3**	SUMO1,PIAS1	0,0	NPJ
**NFYA**	SOX2,E2F4	1,1	PPJ
**CEBP**	SP1,E2F4	0,0	NPJ
**TDGF1**	OCT4,NANOG,SOX2	1,1,1	PPJ
**MYC-SP1**	MYC,SP1	1,1	PPJ
**PIASy**	PIAS1	1	SP
**PBX1**	NANOG	1	SP
**HNF4A**	GATA6,bCAT	1,1	PPJ
**BMP2K**	HNF4A	1	SP
**SP1**	E2F4,SP1,SP3,CEBP,NFYA,MYC-SP1	1,1,0,1,1,0	MJ

[a]1=activation, 0=repression; [b]SP=Single positive influence; SN=Single negative influence; PPJ=Positive plain joint influence; NPJ=Negative plain joint influence; MJ=Mixed joint influence.

2 Novel Qualitative Knowledge-based (Dynamic) Bayesian Network (QK-DBN) Inference

2.1 Qualitative Knowledge (QK)-based (Dynamic) Bayesian network Inference (QK-BN, QK-DBN)

In this section, we formally derive our proposed method of constructing BN and DBN models by utilizing only qualitative knowledge and of making quantitative probabilistic inference. We start our method development by introduction a conventional probabilistic inference problem in the BN and the DBN models. The quantitative training data is denoted by D and the qualitative knowledge is represented by Ω. In the full Bayesian approach, we consider the model's uncertainty in probabilistic inference. In general, we can perform probabilistic inference by model averaging: given evidence E, qualitative knowledge Ω and quantitative observation D, the (averaged) conditional distribution of the remaining variable X is calculated by integrating over the models:

$$P(X|E,D,\Omega) = \int P(X|E,m)P(m|D,\Omega)dm \qquad (10)$$

$$= \int P(X|E,m)P(D|m)P(m|\Omega)dm$$

where P(D|m) is the likelihood of the model and P(m|**Ω**) represents the model's prior probability given the qualitative knowledge.

In QK-BN and QK-DBN, we consider the extreme case of no available quantitative data, i.e. D=null. It is still possible to make Bayesian probabilistic inference of Eq. 10 based on the knowledge **Ω** alone and the evidence E.

$$P(X|E,\Omega) = \int P(X|E,m)P(m|\Omega)dm \quad (11)$$

Each BN or DBN model m is determined by its structure and parameter vector. The Bayesian model space (all possible BN/DBN models) is thus defined by 1) a set of model structures **S**={s_k, k=1,…,K}; 2) for each structure s_k, a continuous ensemble of conditional probability table (CPT) configurations $\widetilde{\boldsymbol{\theta}}_{\boldsymbol{k}}$. The BN/DBN model space can be written as **M**={(s_k, $\widetilde{\boldsymbol{\theta}}_{\boldsymbol{k}}$),k=1,…,K}. For every structure s_k, each possible parameterization in the CPT configuration ensemble $\boldsymbol{\theta}\epsilon\widetilde{\boldsymbol{\theta}}_{\boldsymbol{k}}$ defines a member BN/DBN i.e. m={(s_k,**θ**)|k=1,…,K} and the distribution of a single BN/DBN model is normalized against all models as

$$P(m|\Omega) = P\left(s_{k,}\boldsymbol{\theta}\middle|\Omega\right) = \frac{P(\boldsymbol{\theta}|s_{k,}\Omega)P(s_{k,}|\Omega)}{\sum_{k'=1}^{K} P(s_{k'}|\Omega)\int_{\boldsymbol{\theta}} P(\boldsymbol{\theta}|s_{k'},\Omega)d\boldsymbol{\theta}} \quad (12)$$

where $\alpha = \sum_{k'=1}^{K} P(s_{k'}|\Omega)\int_{\boldsymbol{\theta}} P(\boldsymbol{\theta}|s_{k'},\Omega)d\boldsymbol{\theta}$ is the normalization scalar.

Since a BN/DBN model m is uniquely determined by its structure s and parameter vector **θ**, the BN/DBN model prior probability in Eq.11 can be extended as an integration over the structure space and the structure-dependent parameter space:

$$P(X|E,\Omega) = \int\int P(X|E,m)\frac{P(s,\boldsymbol{\theta}|\Omega)}{\alpha}dsd\boldsymbol{\theta}$$

$$= \sum_{k=1}^{K}\int_{\boldsymbol{\theta}} P\left(X\middle|E,s_{k,}\boldsymbol{\theta}\right)\frac{P\left(s_{k,}\boldsymbol{\theta}\middle|\Omega\right)}{\alpha}d\boldsymbol{\theta}$$

$$= \sum_{k=1}^{K}\int_{\boldsymbol{\theta}} P\left(X\middle|E,s_{k,}\boldsymbol{\theta}\right)\frac{P\left(\boldsymbol{\theta}\middle|s_{k,}\Omega\right)P(s_k|\Omega)}{\alpha}d\boldsymbol{\theta} \quad (13)$$

As we can see from Eq. 13, in order to determine P(m|**Ω**), we need to reconstruct the model structure prior probability $P(s_k|\Omega)$ from the qualitative knowledge **Ω** and the model parameter prior probability $P\left(\boldsymbol{\theta}\middle|s_{k,}\Omega\right)$ given the structure and qualitative knowledge. Without any qualitative information or observation dataset, the (Dynamic) Bayesian network models are uniformly distributed in the model space, i.e. every structure in the (discrete) structure space and every parameter vector configuration in the continuous parameter space are equally probable. It is reasonable to assume that the qualitative knowledge, **Ω**, regarding the network structure is consistent and certain, i.e. expert is fully certain about the dependence and direction of the influential relationships between two variables. Then the probability distribution of the model structure P(s_k|**Ω**) is a Dirac delta function peaked at a specified structure s_k.

$$P(s_k|\Omega) = \delta(s - s_k) \quad (14)$$

Given the k-th model structure, the qualitative information in **Ω** usually does not contain any numerical specification on the parameter configurations since it would otherwise require precise quantitative information on the conditional probability distributions. Instead, the qualitative constraints define a set of possible parameter configurations $\widetilde{\boldsymbol{\theta}}_{\boldsymbol{k}}$. Thusly, the

conditional probability of each parameter vector $\boldsymbol{\theta}$ given the k-th structure and qualitative constraints $P(\boldsymbol{\theta}|s_{k,}\Omega)$ is equal to the probability of this vector belonging to the set of possible parameter configurations $\widetilde{\boldsymbol{\theta}}_{\boldsymbol{k}}$ defined by the constraints in Ω.

$$P(\boldsymbol{\theta}|s_{k,}\Omega) = P(\boldsymbol{\theta} \in \widetilde{\boldsymbol{\theta}}_{\boldsymbol{k}}|s_{k,}\Omega) \tag{15}$$

This conditional probability in Eq. 15 is uniformly distributed for those parameter vectors within the constrained set $\widetilde{\boldsymbol{\theta}}_{\boldsymbol{k}}$ (constrained parameter space), and this probability is equal to zero for those falling outside the constrained parameter space. Namely, given the qualitative knowledge and constraints in Ω and k-th model structure, we take every parameter configuration consistent with the constraints equally in performing the probabilistic inference in Eq. 13.

$$P(\boldsymbol{\theta} \in \widetilde{\boldsymbol{\theta}}_{\boldsymbol{k}}|s_{k,}\Omega) = \begin{cases} 1, & \text{if } \boldsymbol{\theta} \in \widetilde{\boldsymbol{\theta}}_{\boldsymbol{k}} \\ 0, & \text{if } \boldsymbol{\theta} \notin \widetilde{\boldsymbol{\theta}}_{\boldsymbol{k}} \end{cases} \tag{16}$$

Note that it is also possible to use non-parametric Bayesian analysis(7, 8) in combination with qualitative constraints to assign any probability distribution to the conditional probability distribution of a model's structure and parameters in Eq. 14 and Eq. 16, respectively . In this paper, we use the uniform distribution to specify the BN/DBN model structure and parameters' conditional probability distributions in Eq. 14 and Eq. 16, respectively. It is straightforward to show that the normalization factor α in Eq. 12 and Eq. 13 is equal to the size of the constrained parameter space$|\widetilde{\boldsymbol{\theta}}_{\boldsymbol{k}}|$.

$$\alpha = \sum_{k'=1}^{K} \delta(s - s_k) \int_{\boldsymbol{\theta}} P(\boldsymbol{\theta}|s_{k,}\Omega) d\boldsymbol{\theta} = \int_{\boldsymbol{\theta}} P(\boldsymbol{\theta} \in \widetilde{\boldsymbol{\theta}}_{\boldsymbol{k}}|s_{k,}\Omega) d\boldsymbol{\theta} = |\widetilde{\boldsymbol{\theta}}_{\boldsymbol{k}}| \tag{17}$$

Combining Eq. 12, 14, 16 and 17, the conditional probability of a BN model given qualitative knowledge is equal to

$$P(m|\Omega) = P(s_{k,}\boldsymbol{\theta}|\Omega) = \frac{P(\boldsymbol{\theta} \in \widetilde{\boldsymbol{\theta}}_{\boldsymbol{k}}|s_{k,}\Omega)\delta(s - s_k)}{|\widetilde{\boldsymbol{\theta}}_{\boldsymbol{k}}|} = \begin{cases} 1/|\widetilde{\boldsymbol{\theta}}_{\boldsymbol{k}}|, & \text{if } s = s_k \text{ \& } \boldsymbol{\theta} \in \widetilde{\boldsymbol{\theta}}_{\boldsymbol{k}} \\ 0, & \text{if } s \neq s_k \text{ or } \boldsymbol{\theta} \notin \widetilde{\boldsymbol{\theta}}_{\boldsymbol{k}} \end{cases} \tag{18}$$

Eq. 18 means that the models are equally distributed if their structures are consistent with the qualitative information and their parameters fall within the constrained parameter space $\widetilde{\boldsymbol{\theta}}_{\boldsymbol{k}}$.

$$\begin{aligned} P(X|E,\Omega) &= \sum_{k=1}^{K} \delta(s - s_k) \int_{\boldsymbol{\theta}} \frac{1}{|\widetilde{\boldsymbol{\theta}}_{\boldsymbol{k}}|} P(X|E, s_{k,}\boldsymbol{\theta}) P(\boldsymbol{\theta} \in \widetilde{\boldsymbol{\theta}}_{\boldsymbol{k}}|s_{k,}\Omega) d\boldsymbol{\theta} \\ &= \int_{\boldsymbol{\theta}} \frac{1}{|\widetilde{\boldsymbol{\theta}}_{\boldsymbol{k}}|} P(X|E, s_{k,}\boldsymbol{\theta}) P(\boldsymbol{\theta} \in \widetilde{\boldsymbol{\theta}}_{\boldsymbol{k}}|s_{k,}\Omega) d\boldsymbol{\theta} \\ &= \frac{1}{L} \sum_{l=1}^{L} P(X|E, s_{k,}\boldsymbol{\theta}^{\boldsymbol{l}}), \quad \boldsymbol{\theta}^{\boldsymbol{l}} \sim P(\boldsymbol{\theta} \in \widetilde{\boldsymbol{\theta}}_{\boldsymbol{k}}|s_{k,}\Omega) \end{aligned} \tag{19}$$

It is worth noting that, as long as simple inequality constraints are considered as the body of the qualitative knowledge Ω, the problem remains analytically tractable even in higher dimensions. In general, however, integration during Bayesian inference (in Eq. 13) can become intractable by analytical methods. In this case, we employ efficient sampling methods, such as MCMC, to compute the empirical value of the inference in Eq. 19.

2.2 Efficient Monte Carlo Markov Chain

If the local structures of a BN/DBN model (consistent with the prior knowledge) are relatively simple, i.e. the maximum number of parents for all variables in the network are

bounded, then all the local parameters of this model, i.e. $\boldsymbol{\theta}=\{\theta_1,\theta_2,\theta_3,\ldots,\theta_N\}$ are low dimensional. In this case, the intractable integration in Eq. 19 can be approximated by Monte Carlo integration with random sampling within the constraints. Thus, the overall Bayesian probabilistic inference in Eq. 10 can be solved as

$$\begin{aligned} P(X|E,\Omega) &= \int P(X|E,m)P(m|\Omega)dm \\ &= \frac{1}{|\tilde{\boldsymbol{\theta}}_{\boldsymbol{k}}|}\sum_{l=1}^{|\tilde{\boldsymbol{\theta}}_{\boldsymbol{k}}|} P(X|E,m^l) \\ &= \frac{1}{L}\sum_{l=1}^{L} P(X|E,m^l), \qquad m^l \sim P(m|\Omega),\ L = |\tilde{\boldsymbol{\theta}}_{\boldsymbol{k}}| \end{aligned} \tag{20}$$

If the local structures of a valid model are complex, e.g. the maximum number of parents for all variables in the network are unbounded, some (if not all) of the local parameters of this model are high dimensional and the parameter constraints may become more restricted for random sampling in Monte Carlo integration. To efficiently generate samples satisfying the constraints, we exploit a rejection sampling method. The idea is to generate more samples from the current "unexplored" region so that the entire parameter space can be explored evenly. First, we generate samples from the proposed distribution and then reject the samples inconsistent with constraints. The second step is to enhance sampling in the under-sampled space.

Specifically, we define a proposed distribution of the l-th model sample m^l conditioned on the previous samples: $P(m^l|m^{l-1}, \ldots, m^1)$. The more different m^l is from m^1 to m^{l-1}, the higher the probability. We define

$$p(m^l|m^{l-1},\ldots,m^1) \propto \frac{1}{(2\pi\sigma^2)^{N/2}} - q(m^l|m^{l-1},\ldots,m^1) \tag{21}$$

where a kernel density function of Gaussian

$$q(m^l|m^{l-1},\ldots,m^1) = \frac{1}{l-1}\sum_{j=1}^{l-1}\frac{1}{(2\pi\sigma^2)^{\frac{N}{2}}}\exp\{-\frac{\|m^l-m^j\|^2}{2\sigma^2}\} \tag{22}$$

where N is the dimension of the model sample (i.e. number of model parameters, $\boldsymbol{\theta}=\{\theta_1,\theta_2,\theta_3,\ldots,\theta_N\}$) and σ represents the standard deviation. $q(m^l|m^{l-1},\ldots,m^1)$ has high probability in the region close to previous samples. $1/(2\pi\sigma^2)^{N/2}$ is the largest possible value of $q(m^l|m^{l-1},\ldots,m^1)$.

Now we address the problem of how to generate a new sample $\hat{m}^l$ according to this proposed distribution. Our rejection sampling strategy is as follows: 1) We first randomly generate a sample m^l satisfying the constraints; 2) If $l = 1$, this sample is always accepted, otherwise this sample is accepted with the probability $p(m^l|m^{l-1},\ldots,m^1)$. This can be easily implemented as a subroutine: i) Generate a number u from the uniform distribution over [0, 1]; ii) if $u < p(m^l|m^{l-1},\ldots,m^1)$, m^l is accepted; otherwise, it is rejected; 3) If m^l is rejected, go back to Step 1) to generate another sample. Otherwise add the new sample to the sample set $m^l \rightarrow C$; 4) If the size of the samples |C| is smaller than L, then go back to Step 1). We can see that this algorithm includes two rejection steps. Each sample is first tested by the proposed distribution to make it far from previous samples. Then the sample is checked to make sure it satisfies the constraints. Finally, we can get a concise sample set to represent the constraints.

2.3 Probabilistic Inference in Dynamic Bayesian Network

We can generate a sequence of BN/DBN model samples by either simple Monte Carlo integration or more efficient MCMC algorithm presented above depending on the dimensionality of the local structure. All the accepted BN/DBN models follow the probability $P(m|\Omega)$ in Eq.

18. In order to compute the final inference in Eq.19, we need to perform probabilistic inference in each accepted model and calculate their average. Junction Tree (JT) algorithm is usually used to perform belief propagation on static Bayesian networks. For inference in DBN, the naïve approach is to "unroll" the DBN for the desired number of time slices and then perform junction tree inference on the extended model as if it were a static Bayesian network. However, this will be too time-consuming or memory-intensive, particularly in an application with a large junction tree. The interface algorithm uses static junction trees as a subroutine to compute the exact inference in the two-slice temporal Bayesian network (2TBN) and it repeats this inference sequentially over time. In this paper, we utilize the standard interface algorithm(1) to calculate the marginal and joint probability of these nodes in the network under some evidential observation E, i.e. $P(X|E,m^l)$ (see section 1.1.3).

2.4 Belief propagation and Message-passing

After we have constructed the junction tree from the original DBN model, such as Fig. S7, we can perform the standard message-passing algorithm(9) in the junction tree to infer both the joint probability over all variables and the marginal probability of each variable in the network. After message-passing converges and the junction tree becomes a consistent tree(9), we can calculate the joint probability over all variables $\overline{\boldsymbol{X}}$ in the junction tree as

$$P(\overline{\boldsymbol{X}}) = \frac{\prod_i \emptyset_{U_i}}{\prod_j \emptyset_{S_j}} \qquad (23)$$

where $\emptyset_{U_i}$ and $\emptyset_{S_j}$ represent the cluster and sepset potentials respectively. The marginal probability of a variable **X** can be calculated by

$$P(\boldsymbol{X}) = \sum_{\bar{V}\backslash X} \emptyset_U(\bar{V}) = \sum_{\overline{V'}\backslash X} \emptyset_S(\overline{V'}) \qquad (24)$$

i.e. we can pick any cluster **U** or sepset **S** which contains the variable X and integrate out its potential function against other variables in this cluster or sepset.

3. Apply QK-DBN to the curated genetic network

As discussed above, compared to the conventional reverse-engineering approach, this approach does not require a large amount of functional data, which is very limited in hESCs, to train the model. It translates qualitative information curated from the literature of transcriptional regulation and protein-protein interaction into prior distributions of the model's structure and parameters. We then sample all possible models that are consistent with our knowledge by MCMC and use model averaging to infer the final marginal or joint probability. Thusly, our method avoids the possible biased estimation and local maximum when learning from scarce data using the conventional reverse-engineering methods.

In this section, we apply the knowledge-based Dynamic Bayesian network (QK-DBN) method to model the curated genetic network in hESCs. We show here how to use this method to i) predict gene expression level changes upon perturbations; ii) calculate the full energy landscape of cellular states; iii) search for recipes to generate iPSCs and evaluate their reprogramming efficiency; iv) explore reprogramming pathways for cell state transition.

3.1 Gene Expression Changes Prediction in Human ES Cells

The nodes in the constructed genetic network are denoted by **G**={g_1, g_2,…,g_N}, representing the gene expression levels. As discussed before, we assume the nodes in the DBN model are binary nodes which take value of 0 or 1. Value "0" means that this gene is minimally

expressed and "1" means is maximally expressed. The probability of a gene being max-/min-expressed (under condition E) is a continuous value in the range of [0,1]. When a gene is max-expressed, the probability of its node being "1" is 1, i.e. $P(g_i=1|E)=1$. When a gene is min-expressed, the probability of its node being "1" is 0, i.e. $P(g_i=1)=0$. Therefore, we consider this probability positively proportional to the expression level. The higher the probability of $g_i=1$ is, the higher the gene's expression level is.

Definition 3.1 Let $g_{i,max}$ and $g_{i,min}$ represent the maximum and minimum expression level of the i-th gene g_i, respectively. $g_i|E$ is the expression level of g_i under condition E and Δ_i is g_i expression range. The (marginal) probability/belief of g_i being max-/min-expressed is a random value in [0,1] which is linearly proportional to the expression level (intensity) of this node:

$$P(g_i = 1|E) \cong (K_i|E) \times \frac{[g_i|E - g_{i,min}|E]}{g_{i,max}|E - g_{i,min}|E} = (K_i|E) \times \frac{[g_i|E - g_{i,min}|E]}{\Delta_i|E}$$

$$P(g_i = 0|E) \cong (K_i^{'}|E) \times \frac{[g_{i,max}|E - g_i|E]}{g_{i,max}|E - g_{i,min}|E} = (K_i^{'}|E) \times \frac{[g_{i,max}|E - g_i|E]}{\Delta_i|E} \quad (25)$$

K_i and K_i' are unknown or hidden biology-dependent factors for g_i which may affect the proportionality in Eq. 25.

We can further simplify the above equation by rescaling the minimum expression level of g_i to 0 and the expression range to [0, $g_{i,max}|E$]. In this case, the probabilities can be simplified as:

$$P(g_i = 1|E) \cong (K_i|E) \times \frac{g_i|E}{g_{i,max}|E}$$

$$P(g_i = 0|E) \cong (K_i^{'}|E) \times \frac{g_{i,max}|E - g_i|E}{g_{i,\max}|E} \quad (26)$$

Note that the probabilities in the above definitions must satisfy the sum-to-one constraint, i.e.

$$P(g_i = 1|E) + P(g_i = 0|E) = 1 \quad (27)$$

Since $g_{i,max}|E$ and $g_{i,min}|E$ are usually unknown quantities, we cannot directly calculate the absolute expression level under a given condition$g_i|E$. However, it is possible to evaluate the expression level fold-change of a gene across two conditions by comparing the (marginal) probabilities of the gene being max-expressed (or min-expressed) in these conditions. The gene expression ratios between two conditions can be directly evaluated as:

$$ratio_{i,actual} = \frac{g_i|E_1}{g_i|E_2} \quad (28)$$

The gene expression level (intensity) under different conditions $g_i|E_1$ and $g_i|E_2$ can be experimentally measured to evaluate the predicted values calculated by the ratio of $P(g_i = 1|E_1)$ and $P(g_i = 1|E_2)$.

The probability ratios between two conditions (Eq. 26) can be expanded as

$$ratio_{i,probability} = \frac{P(g_i = 1|E_1)}{P(g_i = 1|E_2)} \cong \frac{K_i|E_1}{K_i|E_2} \times \frac{g_{i,max}|E_2}{g_{i,max}|E_1} \times \frac{g_i|E_1}{g_i|E_2}$$

$$= \frac{\alpha_{i,1}}{\alpha_{i,2}} \times ratio_{i,actual} \quad (29)$$

where $\alpha_{i,1}=\frac{K_i|E_1}{g_{i,max}|E_1}$ and $\alpha_{i,2}=\frac{K_i|E_2}{g_{i,max}|E_2}$ are unknown scalars. In Eq. 29, we can see that the ratio between the probabilities is linearly proportional to the ratio between the gene expression levels. E_1 and E_2 are two experimental conditions, such as a control and a knockdown experiment, which are modeled as evidence in DBN. The probabilities can be calculated by Eq. 20. In the case of a knockdown experiment, the evidence in E_2 is represented by clamping specific node(s) (e.g. knocked down genes) to specific expression level(s). Therefore, we can predict the ratios of the gene expressions between knockdown and control experiments by calculating the ratios between the marginal probabilities of this gene under these conditions.

3.2 Energy landscape in the cell state space

In addition to predicting gene expression changes, we can also calculate the full landscape of cell states. In this study, we assume that cell states can be uniquely defined by the expression levels of all genes in the genetic network. We can calculate the potential energy of each state as

$$U_i = -\ln\left(P(S_i)\right) \qquad (30)$$

where $P(S_i)$ is the probability of i-th state of the network and U_i is the potential energy of this state. A collection of the potential energy values of all states in this network can be represented as:

$$\overline{U} = \{U_1, U_2, \dots\dots, U_M\} \qquad (31)$$

where $M=2^N$ and N is the number of genes in the constructed network. We can calculate all potential energy values in $\overline{U}$ from the converged junction tree via Eq. 23, 24 and 30.

To compute the landscape of the genetic network, we need to consider the potentials under all possible (at least most representative) conditions. For our purpose of studying iPSC generation and the differentiation of the hESC, we choose to mimic the most representative scenarios during iPSC generation by varying the expression levels of the three master regulators OCT4, SOX2 and NANOG in hESCs from 0 to 1 with a small interval of 0.2. In DBN inference, for each combination of the levels of these regulators, we clamp their probabilities accordingly and simulate $\overline{U}|E_j$(Energy under j-th condition). Lastly, we calculate and normalize $\overline{U}|E_j$ for all possible j, and then sum them to get the full landscape.

3.3 Searching for recipes to generate iPSC

We want to perform an exhaustive search for reprogramming recipes. As discussed in section 3.1, the predicted (marginal) probability/belief of g_i being max-/min-expressed is linearly proportional to the relative gene expression level (see Eq.29). We use E_1 to denote the hESC state. Since the three master hESC regulators OCT4, SOX2 and NANOG are max-expressed in hESC, without losing generality, we clamp their marginal probability to 1 in our simulation. Then, by QK-DBN inference, we can calculate the marginal probabilities of all the genes in the network in the hESC and these probabilities form a vector of probabilities:

$$\overline{P}_{ES} = \{P(g_1 = 1|E_1), P(g_2 = 1|E_1), \dots\dots, P(g_N = 1|E_1)\} \qquad (32)$$

Similarly, we use E_2 to represent the perturbation conditions specified by an iPS recipe. To search for iPS recipes in our simulation, starting from the differentiation states (OCT4, SOX2 and NANOG initialized to 0), we evolve the DBN given a specific reprogramming perturbation. Consequently, by QK-DBN, for each reprogramming recipe E_2, we can calculate the marginal

probabilities for all the genes in the network given this perturbation. These marginal probabilities under E_2 also form a vector

$$\bar{P}_{recipe} = \{P(g_1 = 1|E_2), P(g_2 = 1|E_2), \ldots \ldots, P(g_N = 1|E_2)\} \quad (33)$$

Based on Eq. 29, since these marginal probabilities should reflect their gene expression levels, we can directly evaluate a recipe by comparing vectors $\bar{P}_{ES}$ and $\bar{P}_{recipe}$. We employ root-mean-square distance (RMSD), Pearson correlation, and Spearman correlation to evaluate the distance from $\bar{P}_{recipe}$ to $\bar{P}_{ES}$.

3.4 Depicting pathways of iPSC generation

Lastly, we explore the cell state transition pathways during reprogramming. As mentioned above, the cell state is defined by the expression levels of all the genes in the network. By DBN definition in section 2.1**,**

$$P(\bar{X}_t) = \prod_{i=1}^{N} \left[P\left(X_t^i \middle| Pa(X_t^i) \right) P(X_{t-1}^i) \right] = \sum_{\bar{X}_{t-1}} P(\bar{X}_t|\bar{X}_{t-1}) P(\bar{X}_{t-1}) \quad (34)$$

where $\bar{X}_t$ and $\bar{X}_{t-1}$ denotes the expression levels of all genes at time t and t-1 respectively. We formulate the probability propagation in DBN for cell states as

$$P(S_t) = \sum_{S_{t-1}} P(S_t|S_{t-1}) P(S_{t-1}) \quad (35)$$

where $S_t = \bar{X}_t$ and $S_{t-1} = \bar{X}_{t-1}$ denote the cell state at time t and time (t-1). The probability of the current cell state is equal to the integration of the product of state transition probability ($P(S_t|S_{t-1})$) and the cell state probability at the last time step ($P(S_{t-1})$). To simplify the computation, we apply maximum-a-posterior (MAP) estimation to predict the state-transition pathway. Namely, at each time step t, we pick the state which maximizes the cell state posterior at the current time step as the current cell state, i.e.

$$\hat{S}_t = \text{argmax}_{\forall s \epsilon S_t}(P(S_t)) \quad (36)$$

Note that the estimated pathway by MAP is not necessarily global maximum.

References:

1. Murphy KP (2002) Dynamic Bayesian Networks: Representation, Inference and Learning. *Ph.D Thesis*.
2. Chang R (2008) Quantitative Inference by Qualitative Semantic Knowledge Mining with Bayesian Model Averaging. *IEEE Transactions on Knowledge and Data Engineering* 20:1587-1600.
3. Chang R, Brauer W, & Stetter M (Modeling semantics of inconsistent qualitative knowledge for quantitative Bayesian network inference. *Neural Networks* 21(2-3):182-192.
4. Alok Aggarwal RJA (1987) A random {NC} algorithm for depth first search. *Proceedings of the nineteenth annual {ACM} conference on Theory of computing*.
5. Gimpel K (2006) Statistical Inference in Graphical Models. *Technical Report* TR-1115.

6. Tian F & Lu Y (2004) A {DBN} inference algorithm using junction tree. *Intelligent Control and Automation, 2004. WCICA 2004. Fifth World Congress on*, pp 4236-4240 Vol.4235.
7. Berger JO (1985) Statistical decision theory and Bayesian analysis. *Springer*.
8. Jordan DMBaMI (2005) Variational inference for Dirichlet process mixtures. *Bayesian Analysis* 1:121-144.
9. Cecil Huang AD (1996) Inference in belief networks: A procedural guide. *International Journal of Approximate Reasoning* 15:225-263.
